# Biosensor-Integrated Microneedle Devices for Diagnosis and Treatment of Chronic and Infectious Diseases: Current Status, Trends and Challenges

**DOI:** 10.3390/bios16040201

**Published:** 2026-04-02

**Authors:** Mohamed M. Ashour, Mostafa Mabrouk, Mohamed A. Aboelnasr, Ahmed M. R. Fath El-Bab, Hanan H. Beherei, Khairy M. Tohamy, Diganta B. Das

**Affiliations:** 1 Biophysics Branch, Faculty of Science, Al-Azhar University, Nasr City, Cairo 11884, Egypt; mohamedashour739@gmail.com (M.M.A.); abomalk3939@gmail.com (M.A.A.); already_a555@yahoo.com (K.M.T.); 2 Refractories, Ceramics and Building Materials Department, National Research Centre, 33 El Bohouth St. (Former EL Tahrir St.), Dokki, Giza 12622, Egypt; hananh.beherei@gmail.com; 3 Mechatronics and Robotics Engineering Department, Innovation School, Egypt-Japan University of Science and Technology (EJUST), Alexandria 71111, Egypt; ahmed.rashad@ejust.edu.eg; 4 Department of Chemical Engineering, Loughborough University, Loughborough LE11 3TU, UK

**Keywords:** microneedle biosensors, interstitial fluid, wearable diagnostics, point-of-care testing, continuous health monitoring, AI-powered diagnostics, personalized medicine, minimally invasive biomarker detection, chronic disease monitoring

## Abstract

Despite advancements in clinical diagnostics, traditional biomarker detection methods (e.g., ELISA) remain limited due to their invasive nature, slow results, and inadequate use for continuous monitoring in low-resource settings. With the rise in chronic, infectious, and metabolic diseases, there is a pressing demand for real-time, minimally invasive diagnostic tools. Nanoengineered microneedle (MN) biosensors offer a promising solution. These painless devices can access interstitial fluid (ISF), a rich source of biomarkers, while utilizing advanced nanomaterials for high sensitivity and multiplexed detection. When combined with AI, IoT connectivity, and cloud-based analytics, MN biosensors enable personalized health data and continuous disease management. This review outlines recent advances in MN technology, including innovations in design and nanomaterial integration, as well as translational challenges like manufacturing scalability and regulatory approval. We explore how MN designs incorporating various sensing modalities can facilitate real-time monitoring of biomarkers such as glucose, lactate, and inflammatory proteins. Importantly, we discuss how these devices can improve healthcare access, reduce costs, and empower patients through everyday monitoring. This review integrates developments in MN engineering with biosensing and therapeutics, positioning biosensor-integrated MNs as pivotal in enabling continuous, minimally invasive disease monitoring and personalized therapy beyond traditional hospital environments.

## 1. Introduction

The skin, the largest organ of the human body, serves as the primary barrier between the internal and external environments. While its primary function is to protect the body from external threats, including pathogens, it also plays a crucial role in regulating body temperature, synthesizing vitamin D, and providing sensory feedback [1]. However, in transdermal drug delivery, the skin’s natural barrier properties, particularly the stratum corneum (the outermost layer of the skin), pose significant challenges. This layer, although essential for skin protective function, acts as a formidable obstacle for the efficient absorption of therapeutic molecules [2]. For patients requiring frequent medication, such as those managing diabetes or chronic pain, this barrier means relying on repeated injections or pills that may not work as effectively as needed.

For several decades, scientists have sought to overcome the stratum corneum’s barrier to enable more efficient delivery of drugs and vaccines. Traditional methods, such as intravenous injections, intramuscular injections and oral medications, while effective, often come with limitations, including pain, poor patient compliance, and systemic side effects [3]. Needle phobia alone affects up to 25% of adults, leading many to delay or avoid necessary treatments. Transdermal drug delivery, on the other hand, offers a non-invasive approach, but it is typically constrained by the skin’s permeability [2]. To address these challenges, a new class of biomedical devices, namely, microneedles (MNs), has emerged as a promising solution. MNs are micro-scale needles, usually ranging in length between 0.1 and 1.5 mm, designed to penetrate the outer layer of the skin (the stratum corneum) without reaching the deeper dermis, where nerve endings and blood vessels reside. This minimal penetration allows for the efficient delivery of therapeutic molecules while minimizing pain and reducing the risk of needle-stick injuries. In practical terms, most users describe the sensation as gentle pressure or a slight scratch, which is far more tolerable than conventional injections. MNs can be used to deliver a wide range of substances, from small-molecule drugs to proteins and vaccines [4]. Over the years, they have shown potential for various applications, e.g., vaccination, biosensing, diagnostic sample collection, and drug delivery [5].

The concept of MN-based drug delivery was first proposed in the early 1990s, and since then, it has evolved rapidly. The versatility of MNs is enhanced by their ability to deliver drugs efficiently through the skin while causing minimal discomfort. These devices can be used for the targeted delivery of drugs, including insulin, hormones, and biologics, with the added benefit of controlled [4]. By bypassing the stratum corneum, MNs allow for the delivery of drugs that would otherwise be poorly absorbed or not absorbed at all through the skin [6]. This opens the door to medications that previously required hospital visits or skilled healthcare providers for safe administration at home. MN are increasingly recognized for their potential to overcome these challenges and provide a more patient-friendly approach to drug delivery [7].

**Figure 1:**
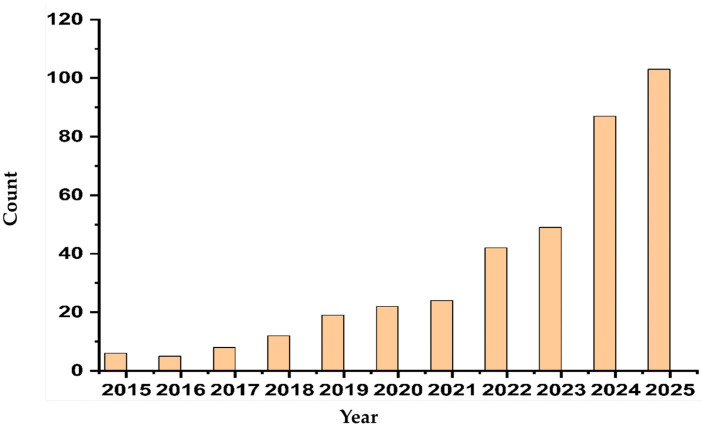
Publication trends related to MN biosensors retrieved from the PubMed database using the search terms: microneedle biosensor, microneedle sensing, and microneedle diagnostics (2015 and 2025).

While MNs themselves provide an innovative method for drug delivery, their potential is significantly enhanced when integrated with biosensors [8], the same platform capable of supporting both sensing and therapeutic delivery. A biosensor is defined as a device that detects the presence or concentration of a specific biomarker and converts this information into a measurable signal. Over the last decade, MN-based biosensors have transitioned from an emerging innovation to a mainstream research focus, as indicated by the increased number of publications in the area (Figure 1). This also reflects a growing recognition that effective treatment depends not just on delivering drugs, but on knowing when and how much to deliver. By incorporating the combination of biosensors with MNs, biosensors provide real-time feedback, enabling the system to monitor biomarkers and trigger drug release based on the specific needs of the patient [8]. For example, glucose-sensitive MNs can deliver insulin in response to fluctuating glucose levels, a breakthrough for diabetes management [9]. For a person living with diabetes, this could mean fewer finger pricks, better blood sugar control, and less worry about dangerous highs or lows throughout the day. This makes these systems suitable for personalized medicine, where treatment can be tailored to an individual’s specific needs, enhancing both efficacy and patient compliance [9].

Biomarker-based drug delivery is revolutionizing how medical treatments are personalized. By detecting specific biomarkers in the body, drugs can be released precisely where and when they are needed, thereby minimising side effects and maximising therapeutic effectiveness [4]. For example, in cancer therapy, specific biomarkers found on or near cancerous cells can trigger the release of targeted therapies directly to the tumour, reducing the impact on healthy cells. This means patients may experience fewer debilitating side effects like nausea, hair loss, and fatigue that often accompany conventional chemotherapy. Similarly, in cardiovascular diseases, MNs integrated with biosensors can monitor heart-related biomarkers and release drugs to prevent a heart attack when necessary. Such systems hold great promise for precision medicine, where treatment is tailored to individual biomarker profiles, offering a more effective, patient-centric approach [10]. These systems hold the key to advancing therapeutic strategies and transforming patient care by enabling real-time monitoring of disease biomarkers and adjusting treatment, which was previously unattainable with conventional drug delivery methods [11].

The aim of this review is to provide a comprehensive and critical overview of biosensor-integrated MN systems for biomarker-guided drug delivery and real-time health monitoring. We examine MN fabrication strategies, material choices, and structural designs, alongside the integration of electrochemical, optical, enzymatic, and piezoelectric biosensors. Particular emphasis is placed on translational relevance, including clinical applications in chronic disease management, oncology, and infectious diseases, as well as emerging closed-loop and AI-enabled systems. Finally, we discuss key challenges and future directions that must be addressed to enable widespread clinical adoption of these technologies. While numerous reviews have discussed microneedle fabrication strategies and transdermal drug delivery systems, relatively few have critically examined the integration of microneedle arrays with biosensing technologies for continuous biomarker monitoring and therapeutic feedback for chronic health diseases. Therefore, this review aims to bridge that gap by examining the convergence of microneedle platforms and biosensor systems for diagnostic and theranostic applications. In addition to evaluating the recent technological advances, the present work highlights the translational landscape of microneedle biosensors, discusses recent human validation studies, and critically discusses the remaining barriers to clinical adoption, including molecular recognition chemistry, sensor stability, and large-scale manufacturing challenges.

## 2. Microneedles

**Figure 2:**
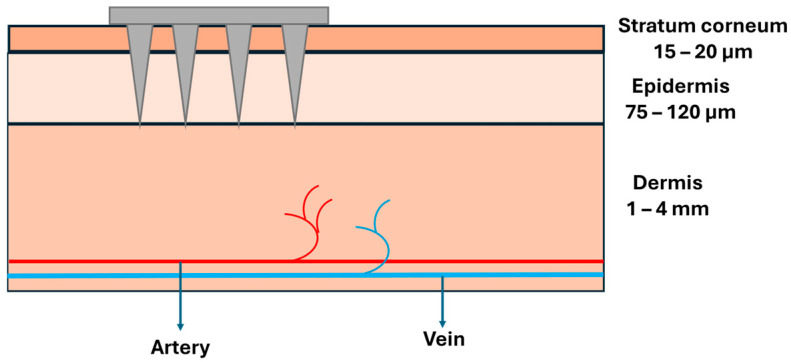
Schematic representation of MN penetration across skin layers.

MNs are engineered microscale structures, typically 0.1–1.5 mm in length, designed to penetrate the skin’s outer barrier, the stratum corneum, while avoiding deeper layers that contain nerves and blood vessels (Figure 2). To put this in perspective, these needles (tip diameter around 42 µm [12]) are shorter than a mosquito’s proboscis, thinner than a human hair, and small enough to be virtually painless. By restricting penetration to this controlled depth, they provide a painless, minimally invasive route for accessing interstitial fluid or for delivering therapeutic agents with high efficiency [13,14]. This careful engineering means patients can receive treatment without the anxiety and discomfort typically associated with traditional needles. In this section, we present a concise overview of MN technologies, with particular emphasis on their design principles, material selection, and fabrication approaches that facilitate safe and minimally invasive penetration of the skin. This section also aims to clarify how specific MN characteristics govern mechanical robustness, user comfort and acceptance, and overall compatibility with integrated biosensing platforms.

### 2.1. Fabrication Techniques of MNs

**Figure 3:**
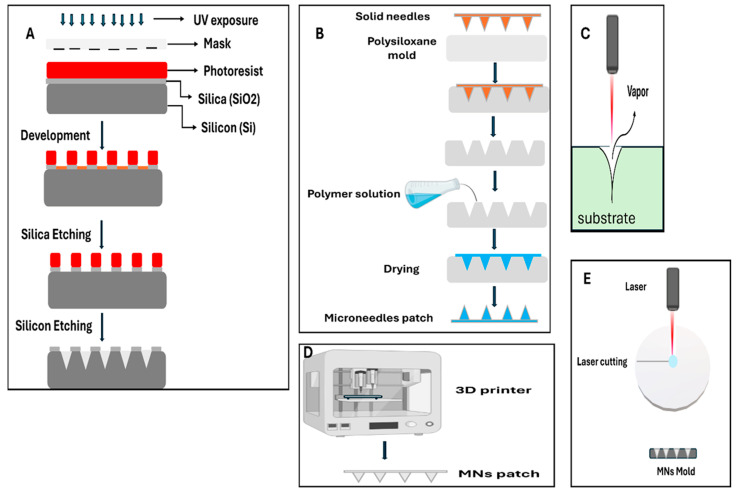
Schematic overview of MN array fabrication methods: (**A**) silicon-based microfabrication involving both photolithography and etching steps; (**B**) micro-molding; (**C**) laser ablation; (**D**) 3D printing; (**E**) laser cutting.

MNs can be fabricated using various methods, each selected based on the material, shape, and medical purpose. Figure 3 illustrates the fabrication techniques used for MNs. With the help of microelectromechanical systems (MEMS), researchers can design highly precise and consistent MNs from silicon, metals, polymers, or ceramics. These can take the form of solid, hollow, coated, or dissolving structures for drug delivery, biosensing, and diagnostic applications. MEMS technology, adapted from the computer chip industry, enables manufacturers to fabricate thousands of identical MNs with precision measured in micrometres, ensuring each device performs consistently. Still, some hurdles remain, such as relatively high production costs, the fragility of certain materials, and the difficulty of integrating these systems for large-scale use that could serve millions of patients worldwide [15,16].

Photolithography is widely used to create detailed patterns, particularly on silicon and polymer surfaces, and it also allows designs to be scaled up through molding. This technique uses light to transfer precise patterns onto a light-sensitive material [17]. Both wet and dry etching techniques offer fine control over the fabrication of high-aspect-ratio structures. More recently, hybrid fabrication approaches have been developed to improve MN tip sharpness and allow the creation of extremely small nanoscale features with dimensions in the nanometer range [18,19].

Laser-based methods, such as ablation and cutting, enable rapid, contact-free fabrication of complex MN shapes from metals, polymers, and ceramics. In this technique, laser-based microfabrication is used to precisely shape microneedle structures by selectively removing material without direct mechanical contact. These techniques are efficient and versatile, but they still face challenges related to heat-induced damage, surface quality issues, and restrictions depending on the material being used [20,21]. Additive manufacturing, particularly 3D printing and two-photon polymerization, provides exceptional freedom in design, making it possible to create hollow or hybrid MN structures and even incorporate bioactive coatings. This is similar to how a 3D printer can create a custom phone case, but at a microscopic scale and with medical-grade precision. With the support of AI-driven design optimization, these techniques open exciting possibilities for customized and multifunctional devices that could one day be tailored to each patient’s unique physiology. However, challenges such as limited resolution, the need for extensive post-processing, and restrictions on suitable materials still need to be addressed [22]. Micro-molding relies on finely crafted master molds to produce uniform and scalable MNs from a wide range of materials. Once a suitable mold is established, microneedle arrays can be replicated in large quantities with high reproducibility and cost efficiency, facilitating scalable manufacturing for clinical applications. This approach is versatile, enabling the creation of solid, hollow, dissolved, or coated designs that can be tailored for applications such as controlled drug delivery and biosensing. Recent advances in biodegradable polymers, nanostructured coatings, and smart MN systems are paving the way for more personalized and minimally invasive healthcare solutions that could eventually be as routine as putting on a bandage [23,24].

From a translational perspective, micro-molding and certain forms of additive manufacturing currently represent the most clinically viable fabrication approaches due to their scalability, reproducibility, and compatibility with biocompatible polymers. In contrast, MEMS-based silicon MNs and high-resolution lithographic techniques, while offering exceptional precision, remain largely confined to laboratory-scale production due to higher costs and brittleness concerns. Laser-based methods occupy an intermediate position, providing design flexibility but requiring careful thermal management to ensure material integrity. Consequently, fabrication strategy selection must balance design complexity with manufacturability and regulatory feasibility.

### 2.2. Types of Microneedles

MNs are classified by their structure, materials, and intended applications. The choice of MN type plays a crucial role in the effectiveness of drug delivery, as well as in patient comfort and compliance. Just as there is no one-size-fits-all medication, different health conditions and patient needs call for different MN designs. The primary types of MNs used in biomedical applications include solid MNs, coated MNs, dissolving MNs, hollow MNs, and hydrogel-forming MNs. Each type has its unique advantages, depending on the therapeutic context and the nature of the drug being delivered [14,25]. Understanding these differences helps explain why some MN patches work instantly while others release medication gradually over hours or days.

The wide range of materials and fabrication techniques highlighted in Table 1 shows how versatile MN platforms have become. Their designs can be tailored for many therapeutic applications, from delivering drugs quickly and locally to providing long-acting systemic therapy or enabling gentle, minimally invasive diagnostic sampling. As these technologies continue to advance, they are steadily moving toward drug delivery systems that are safer, more efficient, and more comfortable for patients.

#### 2.2.1. Solid Microneedles

**Figure 4:**
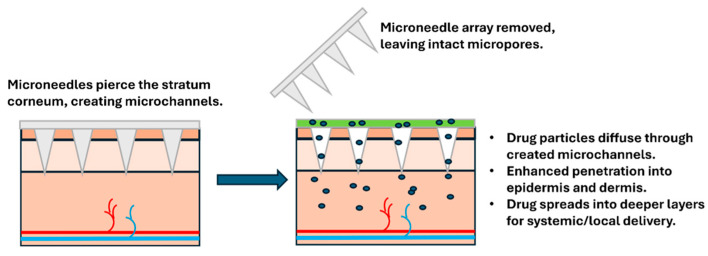
Mechanism of drug delivery using solid MNs. Solid MNs serve as physical permeation enhancers by bypassing the skin barrier and promoting controlled drug diffusion without causing pain or significant tissue damage.

Solid MNs were the first to be developed and are often used in drug delivery systems that rely on a secondary step for drug application. Solid microneedles gently penetrate the stratum corneum, creating small temporary channels that allow medications to pass through the skin more effectively. They consist of solid materials, such as silicon, metals, or polymers, and are designed to create micropores in the skin, allowing drug molecules to pass through these micro-conduits (Figure 4). The poke-and-patch method, wherein drugs are applied after MNs are inserted into the skin, is the most common approach used for solid MNs [26]. In practice, this works much like applying a topical cream after using a skin roller; the MNs create channels, then the medication is applied to the treated area.

The primary advantage of solid MNs lies in their simple design and ease of manufacture. They can be produced using microelectromechanical systems (MEMS) or micro-molding technologies, making them cost-effective and scalable for mass production. This simplicity translates into lower costs and easier quality control, both of which are important when millions of devices must be produced safely and affordably. However, their use is limited by the need for a second step where the drug is applied, which can be inconvenient for patients. For patients suffering a chronic condition at home, having to perform two separate steps, first applying the MNs, then the medication, can feel cumbersome and may reduce how consistently they follow their treatment plan. Moreover, the pores formed by solid MNs typically do not remain open for extended periods, usually closing within a few hours as the skin naturally heals itself, limiting the window for drug delivery [27]. This means timing matters; patients need to apply their medication soon after using the MNs to get the full benefit.

Elim et al. [28] have developed a topical delivery system that combines a thermosensitive nanoemulgel with solid MNs to enhance the skin absorption of β-carotene, the main antioxidant found in *Pandanus conoideus* red-fruit oil. The formulation is built on a red-fruit-oil nanoemulsion, stabilised with polysorbate 80 and Transcutol P, and incorporated into a Pluronic^®^ F127/F68 gel. This gel remains fluid at room temperature but solidifies at normal skin temperature (32–37 °C), allowing the preparation to remain on the skin longer and enhancing drug interaction with the tissue. To further boost drug delivery, the formulation was paired with 1 mm solid MNs, which gently pierce the stratum corneum to create microscopic pathways, an approach widely known to safely improve transdermal penetration. In vitro release experiments showed that the nanoemulgel achieved a β-carotene release of approximately 92%, nearly eight times that of crude red-fruit oil, and followed a Weibull release pattern (R^2^ = 0.9441), indicating a smooth, sigmoidal release profile typical of controlled nanosystems. In ex vivo dermatokinetic tests, the formula, when combined with 1 mm solid MNs, produced a fivefold increase in β-carotene accumulation in skin layers, with faster penetration attributable to MN-created channels. Safety tests demonstrated no hemolysis and no skin irritation, confirming that the system is well-tolerated and suitable for topical use. Altogether, this innovative pairing of a thermosensitive nanoemulgel with solid MNs provides a powerful and safe strategy to significantly enhance the release, penetration, and retention of β-carotene within the skin [28].

#### 2.2.2. Coated Microneedles

**Figure 5:**
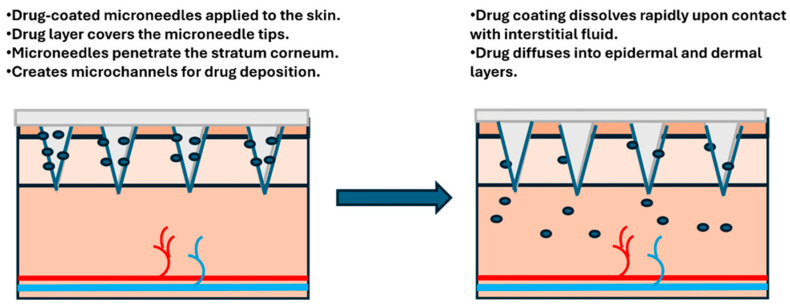
Mechanism of Drug Delivery Using Coated MNs.

Coated MNs consist of solid MNs coated with a drug formulation. In coated microneedles, therapeutic agents are applied as a surface coating that dissolves after skin insertion, enabling rapid drug release into the tissue. The coat-and-poke method involves inserting coated MNs into the skin, where the coating dissolves, releasing the drug directly into the dermis (Figure 5). Coated MNs have gained popularity due to their single-step application and their ability to deliver drugs more efficiently compared to solid MNs [29]. For patients, the simplicity of using this type of MN represents a significant advantage. The application requires only pressing the patch onto the skin, with no subsequent steps to remember or perform. Coated MNs are suitable for delivering small-molecule drugs, proteins, vaccines, and peptides. This versatility has made them particularly attractive for vaccination programs, where a simple stick-on patch could replace traditional syringes, with no need for trained healthcare workers to administer each dose. They can be made from a variety of materials, including polymers, silicon, and metals [30]. The major limitation of coated MNs is the drug loading capacity, which is generally limited to 1 mg. A drug loading capacity of approximately 1 mg is typically sufficient for vaccines and highly potent therapeutics; however, it may be insufficient for drugs that require higher dosage levels. The thickness of the coating and the method of application significantly influence the loading capacity and release dynamics [31]. A precise balance is required, as insufficient coating thickness can limit drug loading and delivery efficiency, whereas excessive thickness may impair dissolution kinetics and compromise the mechanical integrity of the MNs during insertion.

Anbazhagan et al. [30] have developed coated MNs fabricated from biocompatible 3D-printed resin, specifically designed to deliver insulin through the skin in a painless and efficient manner. The MNs array consisted of 100 conical needles (10 × 10), each 1.2 mm in height with an 80 µm tip diameter, engineered to penetrate the stratum corneum smoothly without causing structural damage or discomfort. To convert these solid structures into coated MNs, the researchers used a dip-coating technique in which the MNs were immersed in a formulation containing insulin and polyvinyl alcohol (PVA), which acts as a safe film-forming binder to achieve a uniform drug coating. Careful control over viscosity, immersion time, and withdrawal speed ensured consistent insulin loading on each MN. Penetration tests using parafilm and porcine skin confirmed that the coated MNs were mechanically stable and capable of creating clean micropores suitable for transdermal delivery. In vivo studies showed that once inserted, the insulin coating dissolved within 2 min, rapidly releasing the drug into the dermal microchannels. The coated MNs demonstrated approximately 90% delivery efficiency and elicited a hypoglycemic response comparable to that of subcutaneous injections, maintaining regulated blood glucose levels for several hours. Importantly, skin irritation studies showed that micropores fully closed within 30 min, with no redness or inflammation. In general, these findings highlight coated MNs, formed by 3D-printed resin structures and PVA-based insulin coatings, as a reliable, painless, and clinically promising platform for transdermal insulin delivery [32].

#### 2.2.3. Dissolving Microneedles

**Figure 6:**
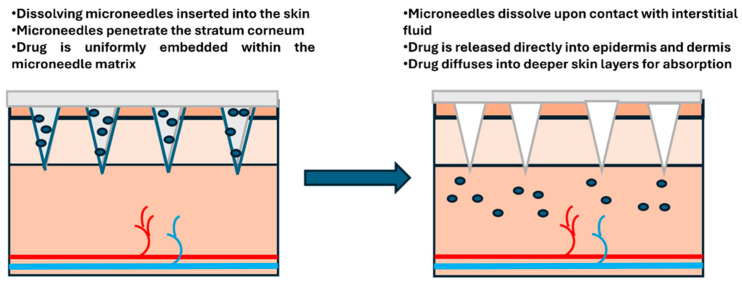
Schematic representation of dissolving MN-mediated transdermal drug delivery.

Dissolving MNs are made from biodegradable materials, such as water-soluble sugars (trehalose, sucrose) or polymers like polyvinyl alcohol (PVA). These materials are typically composed of biocompatible and biodegradable polymers that safely dissolve in the body after administration. The poke-and-dissolve method involves inserting these MNs into the skin, where they dissolve completely, releasing the drug directly into the interstitial fluid (Figure 6). Within minutes to hours, the needles simply melt away, leaving only the medication behind. The key advantage of dissolving MNs is that they eliminate the need for a second application step and leave no residue behind in the skin, reducing the risk of needle-stick injuries and infection [33]. This apply-and-forget approach is particularly appealing for children, needle-phobic patients, and situations where safe disposal of sharps is challenging, like in remote areas or during mass vaccination campaigns.

Dissolving MNs are particularly effective for delivering vaccines, peptides, and biologics. They can accommodate a higher drug loading capacity (up to 33 mg) compared to coated MNs [19]. That is roughly 33 times the amount of medication that coated versions can carry, sufficient to deliver substantial doses of insulin, growth hormones, or other biologics that patients currently inject regularly. However, one challenge with dissolving MNs is their mechanical strength, which can limit their ability to penetrate the skin effectively, especially if the formulation contains hygroscopic materials like sugars [34]. The problem is somewhat paradoxical; the same properties that make these needles dissolve quickly, their ability to absorb moisture, can also make them soft and prone to bending before they pierce the skin, especially in humid conditions or if someone has sweaty hands. Researchers are working to find that point where the needles are strong enough to penetrate reliably but still dissolve completely once inserted [35].

Zong et al. [36] have developed riboflavin-dissolving MNs (RBMNs) as a novel platform for photodynamic eradication of bacterial biofilms, addressing the limitations of conventional antibiotics that struggle to penetrate biofilm structures. The dissolving MNs were fabricated using a pyramidal PDMS mould and composed of PVP/PVA (3:1) blended with riboflavin and nanosilver at the needle tip, while the backing layer incorporated polylysine (ε-PL) to strengthen mechanical durability and provide additional antimicrobial support. The resulting array contained 20 × 20 needles, each approximately 900 μm long and capable of clean, uniform skin insertion. As dissolving MNs, the RBMNs rapidly degraded after insertion, their height decreased by 72% within 8 min, confirming a fast-dissolving behaviour suitable for rapid drug release. Confocal imaging demonstrated that riboflavin diffused deeply into the skin, reaching a penetration depth of approximately 383.93 μm, sufficient to access subdermal biofilms. In vitro release studies revealed that RBMNs released about 97% of riboflavin within 8 min. Altogether, the PVP/PVA-based dissolving MNs, combined with fast (≤8 min) drug dissolution, deep tissue penetration, and photodynamically activated riboflavin, form an effective and minimally invasive strategy for eradicating resistant bacterial biofilms [36].

Recently, silk-based microneedles have attracted increasing attention as a promising biomaterial platform for microneedle fabrication [37]. Silk fibroin represents a highly promising natural biomaterial for the development of next-generation microneedle systems. Its unique combination of biocompatibility, mechanical stability, and controllable degradation enables the fabrication of safe and effective microneedle platforms capable of both therapeutic delivery and biomedical sensing. As research in this area continues to advance, silk-based microneedles are expected to play an increasingly important role in the design of biocompatible, minimally invasive, and multifunctional microneedle technologies for future biomedical and clinical applications [38].

#### 2.2.4. Hollow Microneedles

**Figure 7:**
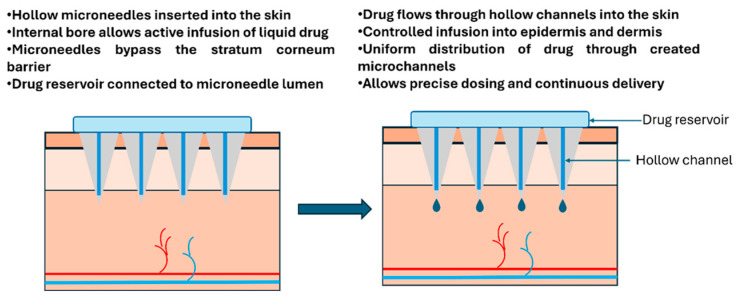
Schematic representation of hollow MN-mediated transdermal drug delivery.

Hollow MNs are characterised by a central microchannel that allows the direct injection of drugs into the skin [39]. The poke-and-flow method involves the insertion of MNs through which drugs can be delivered via a connected reservoir (Figure 7). These MNs are especially useful for the delivery of biological macromolecules and high-volume drugs, as they allow for continuous and precise control over the amount and rate of drug release [40]. For patients requiring extended drug infusions, such as chemotherapy agents or immunosuppressants, this could mean receiving treatment comfortably at home instead of spending hours at a clinic.

**Figure 8:**
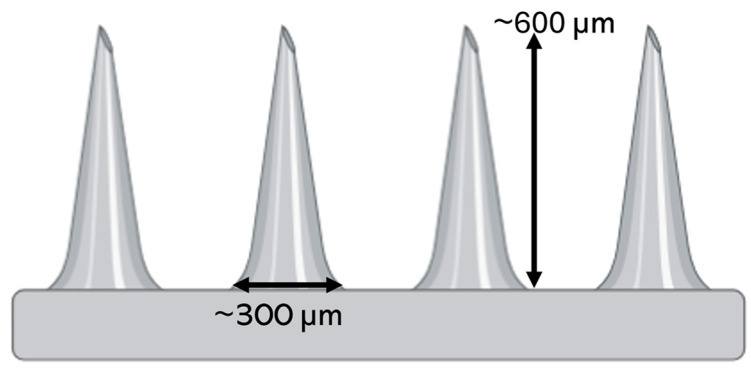
Dimensional characterization of a hollow microneedle array illustrating approximate needle height and needle base diameter.

Hollow MNs offer the advantage of delivering higher doses of drugs compared to other types of MNs, and they are also suitable for fluid-based drug delivery systems. They can handle volumes ranging from microliters to milliliters, making them versatile enough for everything from hormone therapy to emergency medication administration. However, they face challenges such as the risk of blockage or blowout during the injection process, and their fabrication is often more complex and expensive than that of solid or coated MNs [41]. Channel blockage can occur when drug particles accumulate within the narrow lumen of the microneedle or when surrounding skin tissue enters and obstructs the needle opening. A blowout happens when pressure builds up, and the needle separates from its base or the skin seal breaks, causing medication to leak out rather than being delivered. These technical hurdles, combined with manufacturing costs that can be several times higher than simpler designs, have slowed their path from laboratory to pharmacy shelf (see Figure 8).

Jia et al. [42] designed a silk fibroin-based hollow MN system to achieve sustained transdermal delivery of liraglutide, a long-acting GLP-1 analogue used in type 2 diabetes therapy. The hollow MNs were fabricated using silk fibroin (SF) as the structural shell material and hyaluronic acid/liraglutide (HA/LIR) hydrogel as the drug-loaded core. The SF shell, engineered to possess the *Silk I* crystalline form, provides high mechanical robustness, enabling clean penetration of skin while remaining insoluble, swelling to form nanochannels for controlled drug diffusion. The internal cavity, produced by adjusting SF concentration (optimal at 25 mg/mL), allows high drug loading, preserving bioactivity and avoiding unwanted structural transitions. Upon insertion, interstitial fluid enters the cavity, dissolving the HA-encapsulated drug and enabling its diffusion through the swollen silk fibroin nanoporous shell. The release profiles followed Weibull kinetics, consistent with a swelling-controlled mechanism. In vivo pharmacokinetics in SD rats revealed a smooth, sustained plasma profile and relative bioavailability exceeding 80%, demonstrating effective long-term delivery compared to rapid-spike intraperitoneal injection. Overall, the SF/HA-LIR hollow MN platform offers a high-loading, swelling-regulated, sustained-release system capable of delivering up to 0.3 mg per patch, positioning it as a promising non-invasive alternative for long-acting liraglutide therapy [42].

#### 2.2.5. Hydrogel-Forming Microneedles

**Figure 9:**
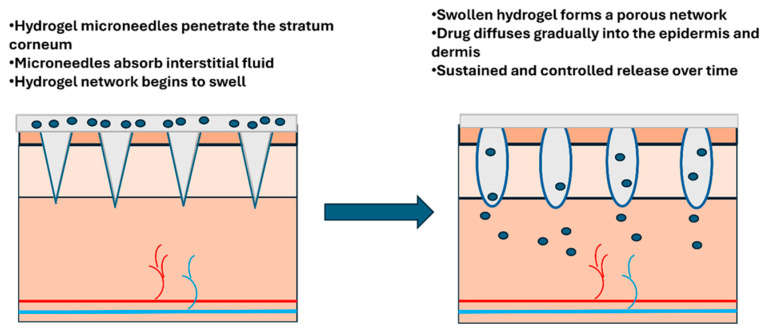
Schematic representation of hydrogel MN-mediated transdermal drug delivery.

Hydrogel-forming MNs consist of an array of crosslinked hydrogels capable of absorbing significant amounts of interstitial fluid, which causes the MNs to swell upon insertion into the skin. The material behaves somewhat like a dry sponge that expands when it contacts water, except in this case, it is absorbing the body’s own fluids. This process creates continuous microchannels that connect the drug reservoir to the underlying tissue, facilitating the delivery of therapeutic molecules. The poke-and-release method involves inserting these MNs into the skin, allowing the drug release rate to be controlled by the rate of hydrogel swelling and dissolution (Figure 9) [43]. As the hydrogel gradually swells and softens, it creates pathways for medication to flow from a patch reservoir into the skin, thereby regulating the rate of drug delivery into the tissue.

Hydrogel-forming MNs are ideal for long-term therapies and can be tailored to provide a controlled release of drugs over time. This makes them particularly promising for conditions requiring steady medication levels over days or even weeks, such as hormone replacement therapy, pain management, or psychiatric medications, where maintaining stable drug concentrations is crucial for effectiveness and preventing side effects. However, their mechanical strength may be insufficient to penetrate the skin effectively, and they may exhibit poor physical stability due to the nature of the hydrogel material [43]. The same softness that allows them to swell and create drug pathways also makes them vulnerable; they can buckle or break during application, especially on calloused or thick skin. Additionally, hydrogels can dry out during storage or degrade over time, which raises concerns about shelf life and whether a patch stored in a patient’s medicine cabinet for months will still work as intended.

Abdelghany et al. [44] have introduced an innovative class of semi-dissolving, hydrogel-forming MNs (ULTRA MAPs) engineered to provide exceptionally long-acting transdermal drug delivery. The MNs were fabricated from a dual-domain polymeric system composed of a covalently crosslinked, water-insoluble chitosan–genipin hydrogel matrix interpenetrated with a water-soluble polyvinylpyrrolidone (PVP) phase. This design allowed the MNs to be produced under mild manufacturing conditions (≤37 °C, hydroalcoholic solvent only) via a vac-and-fill micromoulding method, enabling compatibility with thermolabile drugs. As a model compound, dexamethasone sodium phosphate (DSP), a highly hydrophilic corticosteroid, was incorporated directly throughout the MN tips and baseplate using a one-pot mixed-matrix approach, achieving a high loading of 3 mg per patch. Upon skin insertion, the dry MNs rapidly absorbed interstitial fluid, causing the PVP component to dissolve within minutes, while the crosslinked chitosan–genipin network swelled to form a porous hydrogel that functioned as a sustained-release drug reservoir. In vitro Franz-cell studies demonstrated continuous DSP release for more than 135 days, with 60% cumulative release and measurable output beginning at day 3. Release followed non-Fickian transport, governed by a combination of polymer swelling, erosion, and diffusion, as indicated by the Korsmeyer–Peppas model (n = 0.85, R^2^ = 0.95). Together, these results show that ULTRA hydrogel MNs, built from chitosan–genipin hydrogel and PVP, combine strong mechanical performance, mild fabrication, high drug-loading capacity, rapid hydration, and unprecedented multi-month sustained drug release, offering a powerful platform for long-acting transdermal therapies [44]. In summary, MNs offer a versatile and minimally invasive platform for transdermal access, with tunable mechanical, geometric, and material properties that can be tailored for sensing, drug delivery, or combined theranostic applications. While solid and coated MNs are comparatively mature and easier to manufacture, dissolving, hollow, and hydrogel-forming MNs provide enhanced functionality for controlled release and biomarker-responsive systems. The selection of MN type, therefore, represents a critical design decision that directly impacts clinical feasibility, patient compliance, and integration with biosensing technologies.

**Table 1:** MNs types, fabrication methods and biomedical applications.

**Type of MNs**	**Material**	**Fabrication Method**	**Application**	**Major Outcomes**	**References**
Solid MNs	Maltose	Micromolding	Transdermal drug delivery	MNs dissolved rapidly upon insertion, leaving no residues. Demonstrated safe, minimally invasive, and effective enhancement of systemic transdermal delivery.	[45]
	Silicon	Wet etching	Transdermal drug delivery	Safe, minimally invasive, and effective for enhancing the delivery of hydrophilic drugs.	[46]
	Polylactic acid (PLA)	Micromolding	Transdermal drug delivery	Sharper, thinner MNs reduced puncture force.	[47]
	Fluorescent latex	Wet etching	Transdermal & intradermal delivery of nanoparticles and small drugs	Variability in MN penetration depth, but confirmed the feasibility of nanoparticle delivery via MNs.	[48]
	Polycarbonate	Microfabrication	Transdermal protein delivery	Sharp-tipped MN. Biocompatible, disposable, and safe (no inflammation or allergic reaction in mice). Demonstrated feasibility for vaccine delivery through the skin with engagement of Langerhans cells.	[49]
	Pt-based metallic glass	Thermoplastic drawing of metallic glass	Transdermal drug delivery	High strength, sharp tips, smooth surfaces, and biocompatibility. Demonstrated buckling-assisted hollow formation as a novel fabrication mechanism.	[50]
	Polycarbonate	Microfabrication	Transdermal delivery of hydrophilic molecules	Biocompatible with sharp tips and controlled density/height. High penetration efficacy.	[51]
Coated MNs	Insulin coating with PVA	3D printing	Transdermal insulin delivery	Uniform insulin coating. Rapid drug dissociation. Strong mechanical stability, successful penetration into parafilm and porcine skin. Comparable hypoglycemic effect to subcutaneous insulin injection, stable glucose regulation for hours. Minimal skin irritation.	[32]
	Curcumin-loaded mesoporous silica nanospheres (MSNs) coated with PLGA/PEG polymers	Electrohydrodynamic atomisation (EHDA)	Transdermal delivery of curcumin	Sustained release achieved. MNs pierced the skin without dermal damage.	[52]
	Polyvinylpyrrolidone (PVP)	Micromolding	Intravitreal delivery of dexamethasone	Burst release, Stable drug content. Safer alternative to invasive intravitreal injections, reducing risks of pressure surge and excipient exposure.	[53]
	Hydroxypropyl methylcellulose (HPMC)	3D printing	Transdermal delivery of Donepezil	Uniform arrays with sharp geometry and high mechanical strength. Preserved skin integrity, no apoptosis induction. Safety, rapid initial release followed by sustained release.	[54]
Dissolving MNs	Polyvinylpyrrolidone (PVP) + Polyvinyl alcohol (PVA	Micromolding	Photodynamic therapy (PDT) for bacterial biofilm eradication (*S. aureus*, *E. coli*)	Good mechanical strength. Rapid dissolution. Penetration depth ~384 µm into skin (sufficient for biofilm sites). Reduced inflammation, enhanced collagen deposition, and safe skin regeneration.	[36]
	Polyvinyl alcohol (PVA) + Sucrose	Micromolding	Transdermal delivery of glimepiride	Sharp, mechanically strong MNs. Sustained and complete release. No inflammation. Provided systemic hypoglycemic effect comparable to oral glimepiride but with reduced risks of GI side effects and first-pass metabolism.	[55]
	Sodium carboxymethylcellulose (CMC)	Micromolding	Minimally invasive melanoma therapy (A375 human melanoma cells)	Sharp, uniform MNs with high mechanical strength. Strong antiproliferative and cytotoxic effect due to fucoidan release.	[56]
Hollow MNs	Hyaluronic acid (HA)	Layered mold casting	Sustained and controlled release of liraglutide	High drug loading capacity. Smoother pharmacokinetics with reduced side effects.	[42]
	Borosilicate glass, PVA, HA hydrogel, silicon	3D printing	Transcutaneous infusion of insulin (intradermal vs. subcutaneous delivery)	Predicted drug transport, absorption kinetics, and pain degree index (PDI). Flow rate, needle geometry, and tip opening size critically affect distribution, stress/strain, and pain.	[57]
	Methacrylate hyaluronic acid (MAHA), PVP	Micromolding	Local anesthesia (rapid onset and prolonged effect)	Provided rapid analgesia onset and extended effect up to 48 h. Biocompatible (low hemolysis, no significant inflammation in skin).	[58]
Hydrogel-forming MNs	Polyvinylpyrrolidone (PVP), Chitosan	Micromolding	Transdermal drug delivery	Mechanically robust, penetrated porcine skin effectively. Sustained release. Fabrication compatible with thermolabile drugs (≤37 °C). High drug loading capacity.	[44]
	Poly(methyl vinyl ether-alt-maleic acid) (PMVE/MA)	Micromolding	Ocular drug delivery	Burst drug release (>93% in 2 h), suitable for rapid delivery. Successful scleral penetration, SCS induction, and drug diffusion into the posterior eye segment. Biocompatible.	[59]
	Hyaluronic acid	Micromolding	Therapeutic drug monitoring (TDM) of meropenem via on-patch ISF extraction and near-infrared spectroscopy (NIR)	Strong mechanical strength. Penetrated > 400 µm into porcine skin with 85% success rate. Provided real-time, low-waste, cost-effective TDM for β-lactam antibiotics.	[60]
	Polyvinyl alcohol (PVA), Polyvinylpyrrolidone (PVP)	Micromolding	Transdermal drug delivery for erectile dysfunction (ED), improving SIL solubility and avoiding first-pass metabolism	Strong mechanical strength. High swelling, intact removal with no residue. Sustained release. No hemolysis, no skin irritation; safe and biocompatible.	[61]

## 3. Biosensors Integrated with Microneedles

A biosensor is a device that detects specific analytes by converting their presence into measurable signals. These tools have wide applications, from monitoring diseases and supporting drug development to identifying contaminants, pathogens, or disease markers in body fluids such as blood, urine, saliva, and sweat [62].

This section discusses the integration of biosensors with MN platforms as a strategy for enabling real-time, minimally invasive detection of clinically relevant biomarkers. It highlights how diverse biosensing modalities can be effectively coupled with MN systems to achieve sensitive, selective, and continuous physiological monitoring.

Biosensors stand out for their key advantages, including scalability, ease of use, and the ability to integrate smoothly into point-of-care diagnostic platforms [63]. This means they can work just as well in a village clinic with limited resources as they do in a sophisticated hospital laboratory, a crucial feature for expanding healthcare access globally.

Fundamentally, biosensors combine a biological recognition element, such as enzymes, antibodies, or nucleic acids, with a physicochemical, mechanical, or optical transducer. In this mechanism, the biological recognition element selectively binds to a specific target molecule, while the transducer subsequently converts this binding interaction into a measurable signal. This integration enables the conversion of molecular interactions into measurable analytical signals [64]. Recent progress in electrochemical and optical biosensors, particularly when combined with nanomaterial-based strategies, such as graphene, carbon nanotubes [65], and gold nanoparticles [66], for signal amplification and surface functionalization, has significantly enhanced their sensitivity and performance. These nanomaterials enhance signal amplification, enabling biosensors to detect extremely low concentrations of biomarkers and thereby improving the sensitivity of early disease detection. These advances have led to notable improvements in performance, reduced production costs, and the development of user-friendly device designs, supporting the transition toward decentralised and personalised healthcare [67]. In practical terms, tests that once required expensive laboratory equipment and trained technicians can now be performed with a simple device that costs a fraction of the price and gives results in minutes rather than days.

Progress in nanomaterial engineering, particularly through the design of conductive nanostructures such as graphene, carbon nanotubes, and metallic nanoparticles, has substantially enhanced electron transfer, signal amplification, and overall biosensor sensitivity [68]. These materials conduct electrical signals far better than conventional materials. Advanced surface functionalization techniques now allow for stable and selective immobilisation of biorecognition elements, such as enzymes, antibodies, aptamers, and molecularly imprinted polymers. In essence, scientists can now anchor these detection molecules firmly in place, ensuring they remain active and do not wash away during use, critical for devices that need to work reliably over days or weeks. At the same time, developments in microfabrication technologies, including 3D printing, lithography-assisted patterning processes combined with etching, and laser micromachining, have provided precise control over MN geometry, mechanical strength, and reproducibility [18].

The incorporation of flexible substrates and biocompatible polymers has further enabled the development of minimally invasive, patient-friendly MN designs. These patches can bend with skin movement, are comfortable while wearing, and will not cause irritation or allergic reactions, making them practical for everyday use as well as clinical settings. Collectively, these innovations are reshaping the performance, versatility, and translational potential of MN-based biosensors [69]. The emergence of wearable and implantable biosensors, increasingly enhanced by nanotechnology and artificial intelligence (AI), has made real-time physiological monitoring possible and markedly shortened turnaround times in point-of-care diagnostics [70]. Patients can now wear devices that continuously track their health markers and alert them, or their doctors, to concerning changes before symptoms even appear, shifting healthcare from reactive to proactive. In healthcare, the growing emphasis on continuous biomarker monitoring highlights a broader movement toward real-time physiological surveillance, which represents a critical pillar of precision medicine [71]. Real-time data acquisition enhances diagnostic accuracy, supports timely therapeutic interventions, and facilitates dynamic clinical decision-making, particularly in the management of chronic diseases and in acute care settings [72]. For patients suffering from diabetes, heart disease, or recovering from surgery, this means their care team can spot problems early and adjust treatment immediately, potentially preventing emergency room visits or serious complications that could have been avoided with earlier intervention.

### 3.1. Types of Biosensors

Biosensors can be classified by their transduction mechanism, which determines how the biological signal is converted into a readable output. There are three primary types of biosensors commonly integrated with MNs: electrochemical, optical, and piezoelectric biosensors [73], as discussed below.

#### 3.1.1. Electrochemical Biosensors

**Figure 10:**
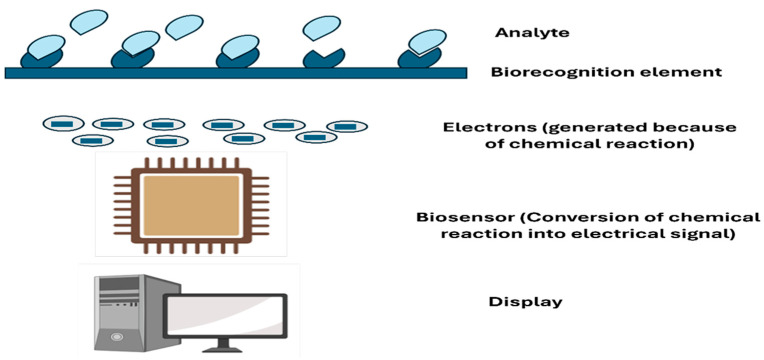
Schematic representation of an electrochemical MN-based biosensing system.

Electrochemical biosensors are the most widely used type of biosensors in MN systems. They are reliable, relatively inexpensive to produce, and work well for the continuous monitoring that patients with chronic conditions need. As shown in Figure 10, these biosensors detect biomarker concentrations by measuring the electrical response generated by the interaction between the biomarker and the sensor’s surface. In essence, when a target molecule such as glucose contacts the sensor, it elicits a small electrical signal. The detection can be based on current, voltage, or impedance changes [74].

One of the main advantages of electrochemical biosensors is their high sensitivity and low cost, making them ideal for continuous monitoring of glucose, lactate, and ions in the body. This sensitivity means they can detect biomarker changes quickly enough to warn someone their blood sugar is dropping before they feel dizzy or confused, potentially preventing dangerous hypoglycemic episodes. The low cost is equally important when devices need to be replaced regularly or used by millions of people with diabetes; affordability can mean the difference between having access to life-changing technology. For example, glucose-sensitive MNs, which incorporate electrochemical sensors, have been developed to deliver insulin when glucose levels rise in diabetic patients [75]. These MNs detect glucose levels in interstitial fluid and trigger insulin release in response to fluctuations, offering a real-time, on-demand solution for diabetes management.

#### 3.1.2. Optical Biosensors

**Figure 11:**
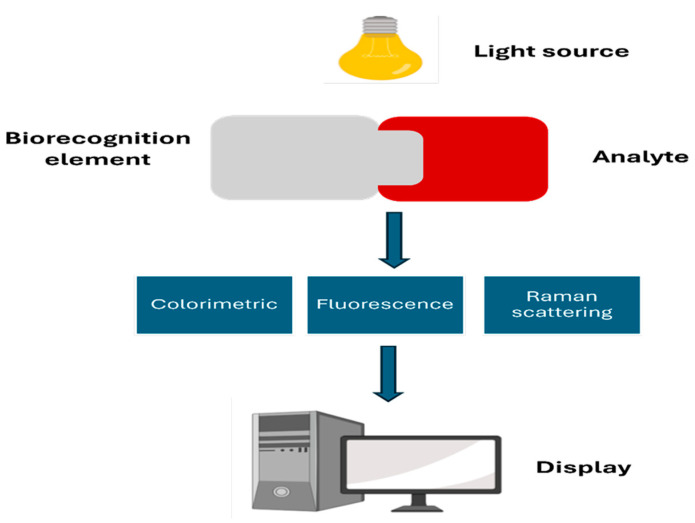
Schematic representation of an optical biosensing system.

Optical biosensors often rely on fluorescence-based detection, which requires an external light source to excite fluorophores and generate a measurable signal. However, other optical sensing approaches are also possible. For example, chemiluminescent reactions produce light directly as a result of biochemical reactions, eliminating the need for external excitation and providing an alternative strategy for optical biosensing [76]. Rather than measuring electrical signals, these sensors monitor changes in how light behaves when it encounters target molecules, much as a prism splits white light into colours when light passes through it. These sensors operate by measuring changes in light transmission, absorbance, or fluorescence that occur when biomolecules bind to the sensor surface (Figure 11) [77]. Each type of light change provides a different window into molecular-level processes, allowing researchers to choose the approach that best suits their specific application.

While optical biosensors are typically less common than electrochemical sensors in MN systems, they offer the advantage of non-invasive detection and the ability to measure multiple biomarkers simultaneously. This multiplexing capability is particularly valuable because many diseases do not present with a single biomarker; being able to track multiple indicators at once provides a more comprehensive picture of what is happening in the body. Recent developments in fluorescence-based optical biosensors have shown potential for targeted drug delivery, such as cancer therapy, where tumour-specific biomarkers can be detected with high selectivity and sensitivity [78]. In this approach, MNs equipped with fluorescence sensors can detect cancer-related biomarkers and release chemotherapeutic agents directly to the tumour site, minimising side effects on healthy tissues. This detect-and-treat capability represents a significant shift from traditional chemotherapy approaches. Instead of loading the body with powerful drugs, the system can identify the problem and deliver medication precisely to that location, sparing healthy cells unnecessary exposure.

#### 3.1.3. Enzymatic Biosensors

**Figure 12:**
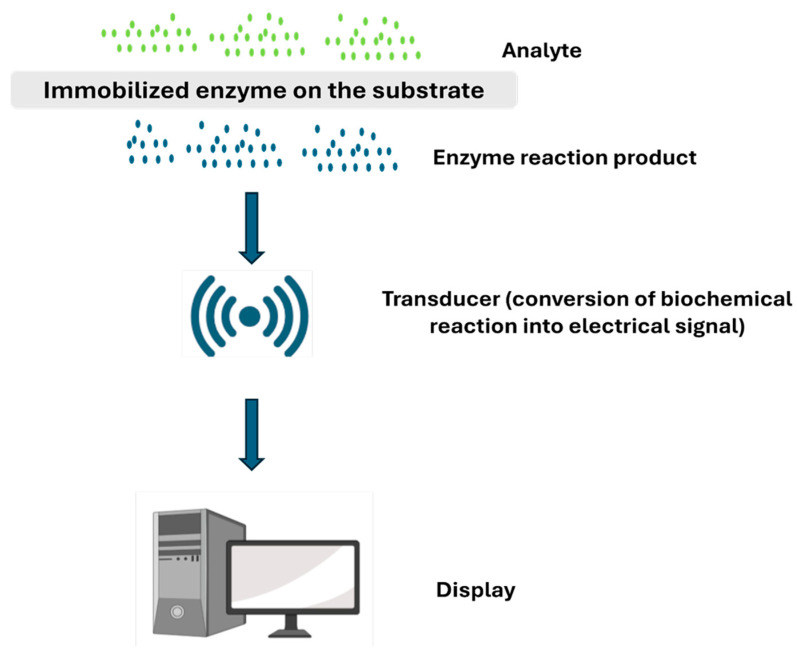
Schematic representation of an enzymatic biosensing system.

Enzymatic biosensors employ enzymes as recognition elements to detect specific biological molecules with high selectivity. Enzymes are evolved to recognise and interact with specific molecules with remarkable precision. Upon binding to their target, the enzyme catalyzes a reaction that generates a measurable byproduct, which is then translated into a detectable signal (Figure 12) [79]. This two-step process, recognition followed by conversion, makes enzymatic sensors both highly specific and relatively easy to measure, since the byproduct often generates a clear electrical or optical signal. A classic example is the glucose oxidase-based biosensor, where the enzyme catalyzes the oxidation of glucose to produce hydrogen peroxide (H_2_O_2_), which is subsequently detected through amperometry measurement [80]. To preserve enzymatic activity and stability over time, enzymes are immobilized onto sensor surfaces through specialized coatings or support matrices. Free-floating enzymes would simply wash away or denature quickly, losing their function within hours. By anchoring them securely to the sensor surface, researchers can extend their working life from hours to days or even weeks, critical for devices meant to monitor patients continuously. This strategy helps maintain sensor performance during repeated measurements and extended use [81]. However, the immobilization process is delicate: attach the enzyme too rigidly, and it cannot move enough to function properly, but attach it too loosely, and it may detach or degrade. Finding this balance is essential for creating sensors that remain accurate and reliable throughout their intended lifespan.

It is important to recognize that many enzymatic biosensors function through electrochemical detection mechanisms. In these systems, the enzyme serves as the biological recognition component, selectively interacting with the target analyte and catalyzing a biochemical reaction. The products of this reaction are then detected by an electrochemical transducer, which converts the biochemical event into a measurable electrical signal. Consequently, enzymatic biosensors are often regarded as a specialized category of electrochemical biosensors, in which enzymatic activity provides molecular specificity while the electrochemical interface enables signal generation and quantification [82].

#### 3.1.4. Piezoelectric Biosensors

**Figure 13:**
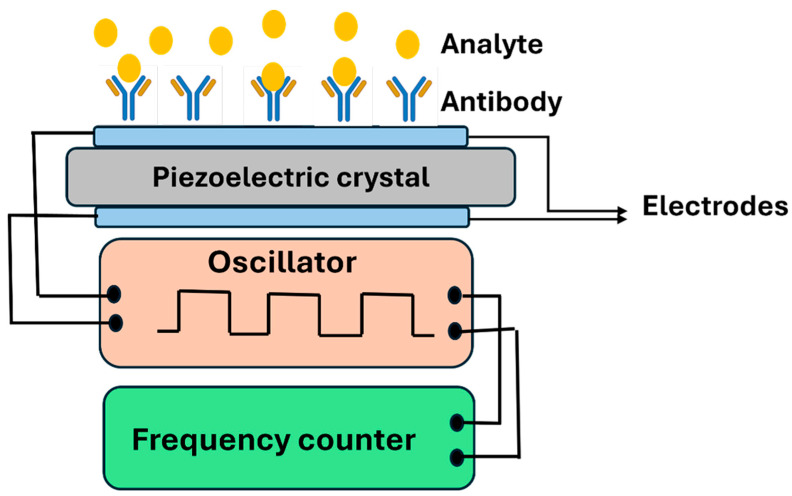
Schematic representation of a piezoelectric biosensing system.

Piezoelectric biosensors measure changes in mass or mechanical stress that occur when a biomarker binds to the sensor. These devices function as highly sensitive mass detectors capable of registering the molecular weight of biomarkers as they attach to the sensor surface, with detection limits reaching the nanogram to picogram range. These changes affect the resonance frequency of the sensor, which can be monitored to quantify the biomarker concentration (Figure 13). The binding of target molecules increases the effective mass of the oscillating crystal, causing a proportional decrease in its resonance frequency, a principle that enables quantitative detection without complex sample preparation.

Piezoelectric sensors are particularly useful for detecting biomarkers at low concentrations and are often used in biosensors for pathogens or disease markers. Their high sensitivity and real-time monitoring capabilities make them an ideal choice for applications such as early disease detection and continuous health monitoring [83]. This capacity for detecting trace amounts of biomarkers during pre-symptomatic or early-stage disease phases addresses a critical clinical need, as timely intervention often significantly improves patient prognosis. In the context of MN systems, piezoelectric biosensors are being explored for applications such as infection monitoring, where the binding of pathogens or disease markers can be detected through changes in the sensor’s frequency.

Piezoelectric biosensors, most commonly quartz crystal microbalance (QCM) devices, have gained attention as affordable, label-free tools that offer both sensitivity and versatility [84]. The label-free detection mechanism eliminates the need for fluorescent tags, radioisotopes, or enzymatic labels, thereby reducing assay complexity, cost, and preparation time while maintaining analytical specificity. They have been successfully applied in medical and environmental settings, detecting targets as varied as hepatitis B virus DNA [85], HIV particles [86]. Beyond infectious diseases, these sensors have shown promise in environmental monitoring, detecting pesticides like paraoxon [87], and in clinical diagnostics, identifying protein biomarkers such as metalloproteinase-9 [88]. The broad applicability across infectious disease diagnostics, environmental analysis, and biomarker detection demonstrates the platform’s fundamental versatility and robust detection principles. One of their strengths is that sensitivity improves dramatically with crystal frequency; for example, moving from 5 MHz to 50 MHz increases mass sensitivity 100-fold [89,90], although higher frequencies come with trade-offs in fragility and complexity [91]. However, higher frequency crystals require thinner quartz substrates, which increases mechanical fragility and handling difficulty during fabrication and operation. This represents a fundamental engineering constraint: optimizing sensitivity necessitates compromises in mechanical robustness and manufacturing complexity. Overall, advances in materials, coatings, and nanotechnology are steadily broadening the potential of piezoelectric biosensors, making them valuable candidates for real-time, reagent-free detection and early disease diagnosis [92]. Continued progress in protective coatings, substrate materials, and microfabrication techniques is progressively addressing these limitations, advancing piezoelectric sensors toward clinical translation and integration into wearable diagnostic platforms.

A recent study highlighted the potential of integrating piezoelectric technology with MN platforms to improve transdermal therapeutic strategies [93]. In this work, researchers developed a piezoelectric-driven MN (PDMN) system in which piezoceramic elements generate acoustic waves that induce controlled cavitation within the dermal tissue. The MN array enables efficient coupling between the device and the skin, allowing deeper penetration of therapeutic agents and improving their local accumulation at the target site. Experimental results demonstrated that this piezoelectric MN platform significantly enhanced the transdermal delivery of photosensitizers and promoted the production of reactive oxygen species during photodynamic therapy. Moreover, both animal studies and clinical trials showed improved treatment efficacy, reduced drug dosage requirements, and shorter treatment durations compared with conventional approaches, while maintaining good patient tolerability. These findings suggest that piezoelectric microneedle systems represent a promising multifunctional approach, combining MN-based transdermal delivery with piezoelectric actuation to enhance therapeutic performance and potentially enable future biomedical sensing and therapeutic applications [93].

Among the biosensor modalities discussed, electrochemical biosensors are currently closest to clinical translation due to their high sensitivity, low power requirements, compatibility with miniaturised electronics, and demonstrated success in continuous glucose monitoring systems. Optical biosensors offer powerful multiplexing capabilities but face challenges related to signal attenuation in skin and system complexity. Enzymatic biosensors remain highly selective but are limited by enzyme stability over long-term use. Piezoelectric biosensors, while exceptionally sensitive and label-free, are still primarily experimental in MN platforms and require further integration studies to justify their advantages over standalone implementations.

## 4. Technological Innovations in Microneedle-Biosensor Systems

The integration of biosensors with MNs represents a significant advancement in biomedical engineering. This section focuses on recent technological innovations that have advanced MN–biosensor systems beyond passive sensing and delivery. It highlights the contributions of smart materials, microelectronics, and system integration strategies to the development of responsive and closed-loop platforms.

By combining the MN’s ability to penetrate the skin with the biosensor’s capacity to detect biomarkers, these systems provide a powerful platform for biomarker-guided drug delivery. Biosensors enable real-time monitoring of the body’s physiological state, enabling targeted drug release in response to specific biomarkers [73,94]. What distinguishes these integrated systems from standalone diagnostics or drug delivery devices is their capacity for closed-loop operation, continuously sensing physiological conditions and autonomously adjusting therapeutic interventions without requiring manual interpretation or intervention.

The development of MN-biosensor systems is a prime example of multidisciplinary innovation. Over the past decade, advances in material science, microfabrication technologies, and sensor technology have contributed to the rapid evolution of these systems [95]. In the late 1990s, reports noted discomfort and dizziness associated with conventional needle-based sampling methods. Since then, MNs have advanced considerably, evolving from simple tools for transdermal drug delivery into sophisticated biosensing platforms capable of monitoring diverse analytes such as glucose, lactate, and various proteins [96]. This evolution reflects a fundamental shift in diagnostic philosophy, from intermittent, clinic-based measurements that provide isolated snapshots of health status to continuous, patient-centered monitoring that captures the dynamic patterns underlying disease progression and treatment response.

It is important to distinguish conventional MN-based drug delivery systems from smart MN platforms. Conventional systems primarily function as passive delivery tools, relying on predefined release kinetics. In contrast, smart MN systems integrate biosensors, signal processing, and in some cases wireless communication, enabling real-time sensing, decision-making, and adaptive therapeutic response. This shift from passive delivery to closed-loop, feedback-controlled intervention represents a fundamental advancement in MN technology.

MN biosensors represent a cutting-edge diagnostic approach, enabling precise molecular analysis through minimally invasive interaction with the skin [97]. These systems combine the minimally invasive sampling ability of MN arrays with the high specificity of molecular recognition elements and the signal transduction mechanisms that define biosensors [69]. Fundamentally, MN biosensors are engineered to breach only the stratum corneum, the skin’s outermost barrier, while avoiding deeper dermal layers that contain pain receptors and blood vessels [98]. This design allows for the painless extraction of interstitial fluid (ISF), which is abundant in clinically relevant biomarkers, or for direct interaction with target analytes within the skin [99]. Importantly, ISF composition closely parallels blood plasma for many key biomarkers, providing clinically meaningful data while circumventing the invasiveness, infection risk, and patient reluctance associated with repeated blood draws. The biosensing element, most often an electrochemical, optical, or enzymatic transducer combined with a biorecognition component such as enzymes, antibodies, or aptamers, is incorporated onto or within the MN structure [100]. When the MN encounters its target biomarker in the interstitial fluid, a specific biochemical reaction is triggered, which is subsequently translated into a quantifiable electrical, optical, or other physical signal [13]. The miniature dimensions of MNs enable localised and continuous sampling, delivering real-time insights into physiological changes. This capability is particularly valuable for chronic disease management, monitoring therapeutic efficacy, and detecting acute physiological events such as hypoglycemia, inflammatory responses, or medication non-compliance before they manifest as clinical symptoms or complications. Furthermore, continuous data streams enable the identification of temporal patterns and trends that single-point measurements cannot reveal, supporting more nuanced clinical decision-making and personalised treatment optimisation. The structural versatility of MNs, spanning solid, hollow, and dissolvable formats, broadens their utility, enabling customized strategies for biomarker detection according to the target analyte and the intended monitoring timeframe [101,102]. This design flexibility allows researchers to match device architecture to specific clinical requirements. Short-term acute monitoring may favor rapidly dissolving formulations, while long-term chronic disease management benefits from stable, reusable electrode-based systems.

### 4.1. Biomarker Detection Mechanisms

The detection mechanism depends on the type of biosensor integrated into the system. In electrochemical biosensors, the biomarker binding to the sensor surface induces a redox reaction that generates an electrical signal, which is proportional to the biomarker concentration. This signal is then used to activate the drug release process [103]. The proportional relationship between biomarker concentration and electrical output enables quantitative monitoring with sufficient precision to distinguish clinically meaningful changes, for instance, differentiating between normal glucose fluctuations and levels requiring therapeutic intervention.

In optical biosensors, the interaction between the biomarker and the sensor surface leads to a change in the absorption or fluorescence of light. This change is measured and used to infer the concentration of the target biomarker [104]. Optical biosensors are particularly useful for multiplexed detection, where multiple biomarkers can be detected simultaneously, providing more comprehensive monitoring of disease states [105]. This multiplexing capability addresses a fundamental limitation of single-biomarker monitoring; many pathological conditions involve complex, multi-factorial changes that cannot be adequately characterized by tracking a single analyte. Simultaneous detection of inflammatory markers, metabolites, and hormones, for example, provides a more complete physiological profile that better informs therapeutic decisions.

Once the biomarker is detected, the information is used to trigger the release of drugs. Several mechanisms can be used to release the drug from the MN, depending on the type of MN and drug. Hollow MNs allow the drug to be injected directly into the skin, whereas dissolving MNs release the drug as the needle dissolves in the skin. In biosensor-integrated MN systems, the release can be triggered by a specific threshold concentration of the biomarker, ensuring that the drug is only released when necessary [106]. This threshold-based activation prevents both undertreatment during periods of genuine therapeutic need and over-treatment when biomarker levels are within acceptable ranges, a critical consideration for medications with narrow therapeutic windows or significant side-effect profiles.

In some cases, the drug release is governed by microfluidic pumps, which are controlled by the signal from the biosensor. This allows for controlled, sustained release of the drug over an extended period [107]. Microfluidic control enables dosing precision unattainable with passive diffusion mechanisms, permitting dose modulation in response to real-time biomarker fluctuations. This dynamic dose adjustment capability is particularly relevant for conditions requiring tight physiological regulation, where static dosing regimens often fail to maintain optimal therapeutic ranges across varying metabolic states and daily activity patterns.

### 4.2. Applications of Microneedle-Biosensor Systems

Compared with conventional diagnostic approaches such as venipuncture-based assays, imaging, or laboratory immunoassays, MN-biosensor systems offer distinct advantages, including reduced invasiveness, faster turnaround times, and the ability to perform continuous monitoring. These features are particularly advantageous in chronic disease management and early disease detection, where intermittent testing may miss critical physiological fluctuations or delay therapeutic intervention. This section provides an overview of the principal biomedical applications of MN-integrated biosensors in the context of chronic disease management, oncology, and infectious disease diagnosis. It aims to highlight the clinical significance and translational promise of these systems by demonstrating their practical advantages over conventional diagnostic and therapeutic approaches in real-world healthcare settings.

The integration of biosensor-enhanced MNs has demonstrated significant potential in various biomedical applications. These systems not only offer an innovative method for drug delivery, but they also enable real-time biomarker monitoring, making them ideal for personalized medicine. This section highlights the key biomedical applications of MN-biosensor systems, including chronic disease management, oncology, and infectious disease detection. Each application is supported by real-world case studies and experimental data, illustrating the capabilities of these systems in improving patient care. These applications represent areas where current standard-of-care approaches face significant limitations, including poor patient adherence to complex dosing schedules, delayed detection of disease progression, and adverse events from non-optimized therapy that biosensor-integrated MNs are uniquely positioned to address.

Together, these advancements show that MN-enhanced biosensors are evolving far beyond their original role in drug delivery. Table 2 shows that MNs are becoming smart diagnostic tools that can monitor important biomarkers continuously and without pain, making them a powerful emerging technology for truly personalized and point-of-care healthcare.

#### 4.2.1. Chronic Disease Management

MNs integrated with biosensors are particularly useful for the management of chronic diseases, such as diabetes and cardiovascular diseases. In diabetes, glucose-sensitive MNs monitor glucose levels continuously in response to changes in glucose concentration [108]. Similarly, in cardiovascular disease, heart-related biomarkers can be detected by biosensors in MNs, enabling on-demand drug delivery to prevent heart attacks or strokes [109]. The chronic nature of these conditions, requiring decades of daily management, frequent monitoring, and precise therapeutic adjustments, makes them particularly suitable candidates for automated, continuous biosensing approaches that reduce the cumulative burden of disease management on patients’ daily lives.

This technology has the potential to replace the traditional methods of glucose monitoring, offering a less invasive, real-time solution for diabetes management. Beyond convenience, continuous monitoring addresses a fundamental limitation of intermittent finger-stick testing, the inability to capture nocturnal hypoglycemia, postprandial spikes, and the dynamic glucose trends that inform optimal insulin dosing, data gaps that contribute significantly to both acute complications and long-term glycemic control challenges.

##### Diabetes and Related Metabolic Disorders

Diabetes, a highly prevalent chronic disease worldwide, often necessitates frequent insulin injections, an approach that can be painful and leads to poor adherence among patients. To address this challenge, researchers have pioneered glucose-responsive MN systems embedded with biosensors that continuously monitor interstitial glucose and enact insulin release when levels rise beyond a threshold. One standout innovation is the bio-responsive core–shell MN patch developed by Wang et al. [110], featuring an H_2_O_2_-sensitive cross-linked gel that triggers insulin release in hyperglycemic conditions, demonstrating highly effective normoglycemia regulation in diabetic animal models. Such innovations represent significant strides toward non-invasive, closed-loop, and patient-adaptive diabetes therapy, aligning with broader efforts in the field to develop smart, responsive insulin delivery platforms [111]. The closed-loop nature of these systems, sensing and responding autonomously without requiring patient intervention, represents a paradigm shift from the constant decision-making burden currently placed on individuals managing diabetes, potentially reducing both the psychological stress and cognitive load associated with chronic disease self-management.

Several credible studies highlight the potential of MN-based sensors for continuous glucose monitoring with wearable integration. For instance, Ju et al. [112] demonstrated a MN array sensor equipped with a wireless transmitter, enabling real-time glucose tracking with improved accuracy [112]. In another example, Sharma et al. [113] reported promising results from a pilot human trial using MN arrays for continuous glucose measurement. These wireless capabilities enable integration with smartphones and cloud-based analytics, allowing not only real-time patient monitoring but also data sharing with healthcare providers for remote intervention and trend analysis, features particularly valuable for vulnerable populations, including elderly patients, children, and those in geographically isolated areas.

**Figure 14:**
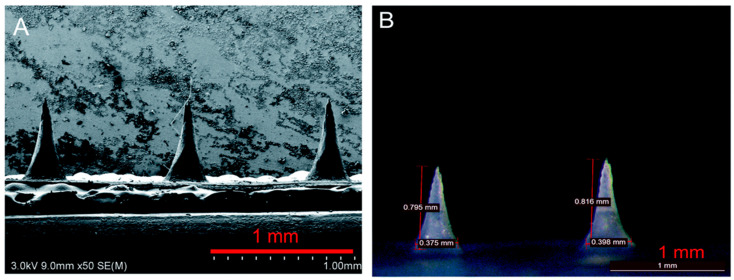
Optical and scanning electron microscopy (SEM) images of the silk/D-sorbitol MN electrode (silk/D-sorbitol ratio = 10:3): (**A**) SEM image of the MN electrode; (**B**) optical image of the MN electrode. Reprinted with permission from ref. [114]. Copyright 2020 Royal Society of Chemistry.

A silk/D-sorbitol MN electrochemical biosensor incorporating glucose oxidase (GOD) was developed for minimally invasive continuous glucose monitoring. The MNs, averaging 800 μm in length with a breaking strength of 0.8–1.4 N, effectively penetrated the skin while remaining stable in interstitial fluid. The sensor displayed a strong linear correlation with glucose across the physiological range of 1.7–10.4 mM (R^2^ = 0.996), producing rapid and reproducible amperometric responses. Importantly, it retained over 93% of its activity after 24 h of continuous use and maintained 97.7%, 91.3%, and 76.9% of its initial signal after 35 days of storage at 4, 25, and 37 °C, respectively [114] (see Figure 14). This extended shelf stability across varied storage temperatures addresses practical deployment considerations; devices must remain functional throughout supply chain distribution, pharmacy storage, and home medicine cabinet conditions, requirements often overlooked in laboratory-stage development but critical for real-world accessibility.

Among the latest innovations, highly porous gold (h-PG) MN biosensors have shown excellent potential for minimally invasive glucose monitoring. By creating a porous gold surface with nearly a 100-fold increase in electroactive area compared to bare MNs, the device achieved remarkable sensitivity, recording 50.86 µA cm^−2^ mM^−1^ with a broad linear range of 0.1–10 mM glucose and a low detection limit of 50 µM. The sensor maintained stability with only about 20% signal loss after 30 days of continuous use, while also demonstrating high selectivity, with interfering molecules such as fructose, mannitol, and galactose showing negligible responses (ascorbic acid gave only ~10% of the glucose signal). Importantly, continuous monitoring in a hydrogel-based skin model confirmed robust operation, with a realistic 2 min lag time yet consistent detection across physiological glucose levels [115]. The 2 min lag between interstitial fluid and blood glucose represents a clinically acceptable delay for most diabetes management scenarios, though it necessitates predictive algorithms for detecting rapid glucose changes, a consideration particularly relevant for preventing exercise-induced hypoglycemia or managing postprandial excursions.

Recent progress has also enabled MN arrays to monitor multiple metabolites simultaneously, broadening their clinical applications. A gold MN array modified with multiwalled carbon nanotubes, polymethylene blue, and enzyme coatings was developed for dual lactate and glucose sensing in artificial interstitial fluid. The lactate biosensor exhibited a very high sensitivity of 797.4 ± 38.1 µA cm^−2^ mM^−1^, with a linear range of 10–100 µM and a detection limit of 3 µM, while the glucose biosensor achieved 405.2 ± 24.1 µA cm^−2^ mM^−1^ sensitivity across 0.05–5 mM with a detection limit of 7 µM. Both platforms retained over 80% of activity after 30 days of continuous use and showed strong selectivity, remaining unaffected by interfering molecules such as uric acid, ascorbic acid, or hydrogen peroxide. In a hydrogel skin model mimicking physiological conditions, the system reliably detected lactate concentrations of 12 mM following simulated exercise and glucose levels of 10 mM after a meal, with only a 2 min response lag. These findings highlight the potential of MN biosensor arrays as minimally invasive, real-time wearable tools for integrated metabolic monitoring in both clinical care and athletic performance [116]. Simultaneous lactate–glucose monitoring addresses metabolic complexity that single-analyte systems cannot capture. Lactate elevation may indicate impaired tissue perfusion, sepsis, or intensive exercise, while concurrent glucose data helps distinguish between exercise-induced metabolic changes and pathological states, information critical for appropriate clinical interpretation and intervention decisions.

A MN system coated with PVDF-Nafion nanomembranes was tested for long-term implantable glucose sensing. The device achieved a linear detection range up to 20 mM glucose with strong signal reproducibility and minimized biofouling effects due to its selective membrane. In vivo studies confirmed accurate tracking of blood glucose fluctuations with stable responses over several days, demonstrating its ability to reduce signal drift and tissue interference compared to conventional sensors [117]. Biofouling, the accumulation of proteins, cells, and biomolecules on sensor surfaces, represents one of the most persistent challenges in long-term implantable devices, progressively degrading signal quality and necessitating frequent replacement. Anti-fouling membrane strategies that extend functional lifetime directly translate to reduced replacement frequency, lower healthcare costs, and improved patient acceptance.

MN sensing platforms are also being extended to stress biomarkers, with cortisol serving as a prime example. Zhou et al. [118] have designed an aptamer-functionalized MN patch integrated with hybridization chain reaction (HCR) amplification to capture and quantify cortisol directly from interstitial fluid. The patch, composed of methacrylated hyaluronic acid, demonstrated robust skin penetration (~380 μm) and excellent biocompatibility, with HaCaT cell viability remaining above 95%. Under optimized conditions, the device achieved a detection limit as low as 0.048 μM, covering physiological cortisol concentrations in ISF. In vitro assays showed a strong linear fluorescence response across 0–0.5 μM cortisol (R^2^ = 0.9992), while selectivity tests confirmed negligible interference from structurally related steroid hormones. In vivo, the patch successfully distinguished cortisol fluctuations in mice, detecting elevated levels (~127 nM) after exercise-induced fatigue compared to ~75 nM at rest, results that closely mirrored standard ELISA measurements [118]. Cortisol monitoring extends MN applications beyond metabolic disorders into stress physiology, mental health assessment, and endocrine dysfunction diagnosis, domains where current diagnostic approaches rely on invasive blood draws or imprecise salivary measurements that poorly capture the dynamic circadian and stress-responsive nature of cortisol secretion.

Clinical validation of MN biosensors is emerging, with lactate as a key example. In the first-in-human trial by Ming et al. [119], the results provide compelling evidence that MN biosensors can deliver accurate, minimally invasive, real-time lactate monitoring in humans. A solid MN patch was applied to the forearms of five healthy adults undergoing 30 min of cycling exercise, followed by recovery. The patch continuously measured interstitial fluid lactate and showed close agreement with venous blood and microdialysis, tracking levels from baseline (~1.4 mmol/L) to peak exercise values of up to 13.0 mmol/L. Importantly, the device exhibited only a median lag time of 5 min (IQR −4 to 11 min) relative to blood, capturing both rises during exertion and clearance during rest. The system was well tolerated, with minimal discomfort (mean score 0.4/10) and no adverse events, and participants strongly preferred it over repeated blood sampling [119]. This human validation study addresses a critical translational gap: many promising laboratory technologies fail when deployed in real physiological conditions due to unforeseen biological variability, motion artifacts, or poor patient tolerance. Demonstrating clinical accuracy, acceptable lag times, and strong patient preference provides essential evidence supporting regulatory approval pathways and clinical adoption.

**Figure 15:**
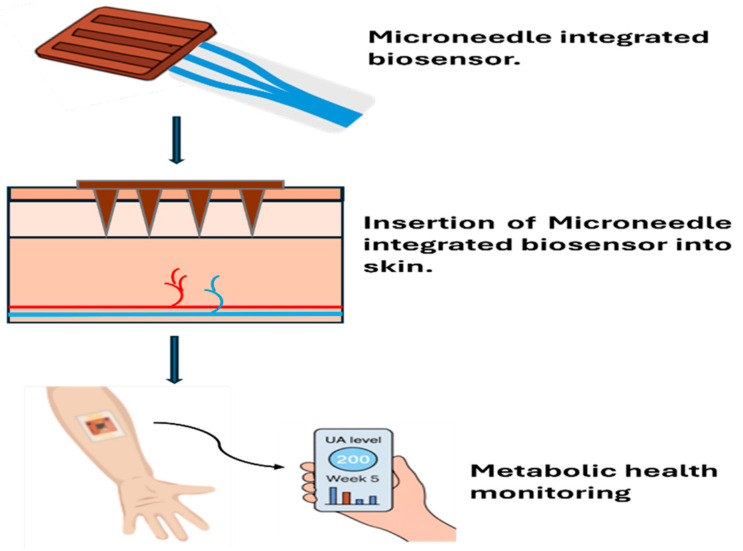
Schematic representation of a wearable MN-based electrochemical platform for continuous uric acid monitoring.

MN-based sensing has also been extended to uric acid, a critical biomarker for gout, renal function, and metabolic health. As shown in Figure 15, this study, a low-cost, fully 3D-printed MN array sputtered with nanostructured gold was developed as a wearable electrochemical patch for continuous ISF monitoring. The MNs, averaging ~860 μm in height and ~30 μm tip diameter, demonstrated reliable skin penetration with minimal deformation, showing only an 8.4% signal decrease after four repeated insertions. Electrochemical tests revealed excellent sensitivity of 25 nA μM^−1^, with linear detection across 50–500 μM and a low limit of detection of 19 μM, well within the physiological uric acid range. The device maintained repeatability with relative standard deviations below 2% over 26 cycles and preserved its performance in protein-rich solutions due to the antifouling properties of the nanostructured surface. Ex vivo experiments through porcine skin confirmed reversible and stable detection with sensitivities around 9.2 nA μM^−1^, while preliminary on-body testing in humans measured uric acid levels of ~194 μM, closely aligning with physiological values [120]. The 3D-printing fabrication approach represents a significant advancement toward cost-effective, scalable production, potentially reducing manufacturing costs by an order of magnitude compared to traditional lithographic methods while enabling rapid design iterations and customization for different patient populations or anatomical sites.

##### Cardiovascular Diseases

Cardiovascular diseases are another major cause of morbidity and mortality worldwide. Early detection of biomarkers, such as troponin, brain natriuretic peptide (BNP), and C-reactive protein (CRP), is crucial for early intervention. The clinical challenge lies in the narrow temporal window for effective intervention; cardiac troponin elevation begins within 2–4 h of myocardial injury, yet traditional diagnostic pathways, involving emergency department presentation, blood draw, laboratory processing, and result reporting, often require several hours, potentially delaying time-sensitive treatments such as percutaneous coronary intervention or thrombolytic therapy. MN-biosensor systems can be used to detect these biomarkers in real-time, triggering drug delivery in response to acute events such as heart attacks or stroke. This capability is particularly valuable for high-risk populations with recurrent cardiac events, unstable angina, or post-myocardial infarction patients, where continuous surveillance could enable prophylactic intervention before symptoms manifest or irreversible tissue damage occurs.

A 2025 study by Mirzajani et al. [121], introduced MiCaP, a MN-based capacitive biosensor capable of minimally invasive, in situ monitoring of cardiac troponin I, a key biomarker of myocardial injury, demonstrating the technical feasibility of MN-based troponin sensing. Although this prototype does not yet include drug release, it lays a critical foundation for future integrated theranostic systems where detection can directly trigger localized therapy [121]. The progression from sensing-only platforms to fully integrated theranostic devices represents a logical developmental trajectory. Establishing reliable biomarker detection in physiological conditions is a prerequisite before incorporating the additional complexity of controlled drug release mechanisms, regulatory pathways, and safety validation required for autonomous therapeutic systems.

The polylactic acid MNs coated with CRP-specific aptamers for cardiovascular risk assessment through the detection of CRP were fabricated with precise dimensions (height ~2340 μm, tip diameter ~258 μm) to ensure reliable dermal penetration, confirmed in mouse models at an average depth of 463 ± 67 μm. The system enabled selective CRP capture, showing high specificity against related biomarkers such as NT-proBNP, troponin I, and fibrinogen. Quantitative assays demonstrated a strong linear response across clinically relevant CRP concentrations of 0–10 mg/L, with limits of detection as low as 0.1 mg/L in PBS and 0.5 mg/L in blood (R^2^ = 0.96–0.98). Optimization studies revealed that an array of eight MNs with a 10 min incubation produced the most robust signals. Despite reduced capture efficiency in whole blood compared with PBS, the device consistently detected physiologically relevant CRP levels, aligning with cardiovascular risk thresholds (<1, 1–3, >3 mg/L). These results emphasize the potential of aptamer-functionalized MNs as minimally invasive, point-of-care tools for early cardiovascular disease risk monitoring and proactive health management [122]. The ability to stratify patients according to established clinical risk thresholds directly from interstitial fluid represents a significant advancement over current practice, where CRP testing requires venipuncture and laboratory analysis. This could enable routine cardiovascular risk screening in primary care settings, pharmacies, or even home monitoring for individuals with family history or multiple risk factors, potentially identifying at-risk individuals before they experience clinical events.

MN biosensors are also being applied to lipid monitoring, with cholesterol emerging as a critical target. Li et al. [123], has fabricated and functionalized a hollow MN array integrated with platinum and silver wires with cholesterol oxidase for real-time electrochemical detection. The sensor exhibited a high sensitivity of 0.201 mA mM^−1^ with excellent linearity (R^2^ = 0.991) across 1–20 mM in PBS, well within the pathological and physiological cholesterol range. In artificial interstitial fluid, the device maintained strong performance with a detection limit as low as 0.5 mM and linear correlation (R^2^ = 0.999). Tests in a skin-mimicking phantom gel further validated its practicality, showing robust linearity across 2–10 mM cholesterol (R^2^ = 0.9996). The biosensor demonstrated excellent selectivity, with negligible responses to glucose, lactate, uric acid, and ascorbic acid, and retained 86% of its activity after four weeks of storage at 4 °C. Collectively, these findings highlight the feasibility of hollow MN platforms for minimally invasive, accurate, and durable cholesterol monitoring, supporting future translation into wearable systems for cardiovascular risk management [123]. Continuous or frequent cholesterol monitoring addresses a limitation of current lipid management, and single-point measurements provide only snapshots that may not reflect dietary responses, medication adherence, or the effectiveness of lifestyle interventions. Dynamic cholesterol tracking could enable personalized titration of statin therapy, real-time feedback on dietary choices, and early detection of treatment non-response, transforming lipid management from periodic laboratory assessments to continuous metabolic surveillance.

**Figure 16:**
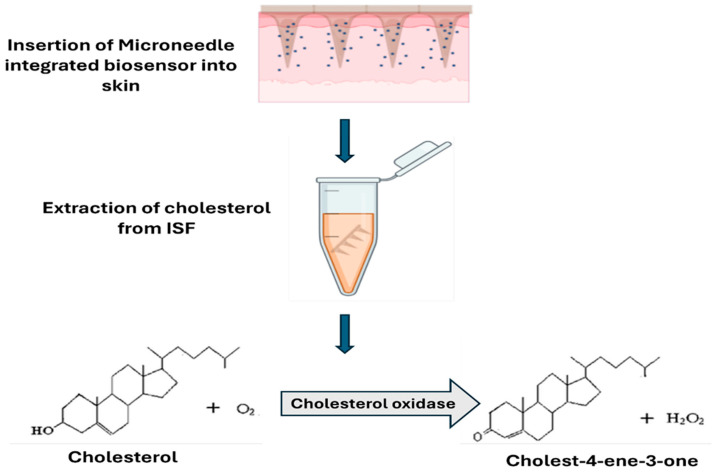
Schematic representation of a cholesterol biosensing system utilizing interstitial fluid extracted by MNs.

Hydrogel MNs are increasingly enabling non-invasive cholesterol monitoring: fabricated from 30% acrylamide, they achieved effective porcine skin penetration and extracted up to 23 μL of interstitial fluid within 60 min, sufficient for electrochemical testing. The integrated biosensor, featuring a graphene-modified electrode with a polyaniline interlayer for cholesterol oxidase immobilization, delivered exceptional analytical performance. It detected cholesterol with a limit of detection of 0.15 mg/dL in PBS and 0.2 mg/dL in bovine serum, offering a linear range of 0.3–30 mg/dL (R^2^ = 0.97). Compared with a commercial cholesterol biosensor, the device, as shown in Figure 16, has achieved markedly higher sensitivity and accuracy, capturing reliable signals even at low concentrations. Stability testing confirmed less than 5% signal deviation after two weeks of storage at 4 °C, while selectivity assays showed interference from glucose, uric acid, ascorbic acid, sucrose, and BSA altered responses by less than 10%. Importantly, trials using agarose skin-mimics and porcine skin demonstrated a nearly perfect correlation (slope ~ 1.0; R^2^ = 0.99) with standard enzymatic assays, confirming its reliability for real-world application. This study underscores the feasibility of MN-assisted ISF extraction combined with graphene-based electrochemical sensing as a minimally invasive, accurate, and stable strategy for cholesterol monitoring [124]. The demonstrated correlation between MN-derived ISF measurements and standard blood-based assays is crucial for clinical acceptance. Physicians and patients require confidence that ISF cholesterol levels reliably reflect systemic lipid status and can guide treatment decisions with equivalent accuracy to established laboratory methods. This validation bridges the gap between promising technology and clinical utility, addressing regulatory requirements for analytical equivalence that must be met before widespread clinical adoption.

#### 4.2.2. Cancer Diagnosis and Therapy

Another application of MN-biosensor systems is in oncology, where the detection of cancer biomarkers can trigger the release of targeted therapies. These systems are particularly useful for early cancer detection, where they can detect tumor markers such as PSA (prostate-specific antigen), epidermal growth factor receptor (EGFR), and cancer antigen 125 (CA125) [5], and release chemotherapeutic agents directly to the tumor site [125]. The paradigm shift toward early detection is driven by compelling survival data; five-year survival rates for most solid tumors exceed 90% when diagnosed at localized stages but drop below 30% after metastatic spread, underscoring the critical importance of technologies capable of identifying malignancies during their most treatable phases. This localized approach minimizes the impact of chemotherapy on healthy tissues, reducing side effects and improving therapeutic outcomes. Beyond improved survival, localized delivery addresses quality-of-life concerns that significantly affect treatment adherence, systemic chemotherapy-induced alopecia, severe nausea, neutropenia, and fatigue often lead patients to delay, refuse, or discontinue potentially curative treatments, outcomes that targeted delivery systems could substantially mitigate.

Wang et al. [126] developed multifunctional inverse-opal MN arrays capable of fluorescence-enhanced detection of tumor biomarkers, including PSA in interstitial fluid. Upon biomarker recognition, these patches also enabled controlled, localized drug release directly at the tumor site, enhancing therapeutic efficacy while reducing systemic side effects [126]. This integration of diagnostic and therapeutic functions within a single platform exemplifies the theranostic concept; the same device that identifies disease presence and quantifies its severity can autonomously initiate treatment, eliminating the temporal gap and logistical complexity inherent in separate diagnostic and therapeutic procedures.

The fluorescence-based biosensors can detect these biomarkers with high sensitivity and trigger the release of chemotherapeutic agents directly to the tumor site, improving the efficacy of treatment while minimizing damage to healthy tissues [5]. Fluorescence detection offers particular advantages in oncology applications; it enables multiplexed detection of multiple tumor markers simultaneously, supports quantitative assessment of biomarker concentrations to guide dose optimization, and can potentially distinguish between benign and malignant conditions based on biomarker expression patterns, capabilities that single-analyte electrochemical approaches cannot readily provide.

Recent advances have demonstrated dissolvable MN patches capable of delivering chemotherapeutics such as doxorubicin and docetaxel directly into tumor tissue, achieving significant tumor regression in breast cancer models while minimizing systemic toxicity [31]. The use of dissolvable formulations addresses a practical concern in cancer treatment. Patients with advanced disease often experience cachexia, immunosuppression, and compromised wound healing that increase infection risk from retained foreign materials. Completely biodegradable delivery systems eliminate this concern while simplifying patient management.

MN systems have shown promise in reducing the systemic side effects commonly associated with chemotherapy, such as nausea and fatigue, by delivering drugs directly to tumour sites and minimising exposure to healthy tissues. Studies and reviews indicate that MN-mediated transdermal delivery enhances precision and efficiency, leading to reduced systemic toxicity and improved therapeutic safety and outcomes [127]. Quantifying this benefit clinically remains challenging, while preclinical models demonstrate clear reductions in systemic drug exposure, translating these findings to human patients requires accounting for tumor heterogeneity, variable skin properties, individual pharmacokinetics, and the complexity of multidrug regimens, factors that will require carefully designed clinical trials to definitively establish therapeutic advantages.

MN biosensors are also being tailored for protein biomarkers, with vascular endothelial growth factor (VEGF) serving as a critical target in angiogenesis-related diseases. Flexible PLA MNs were integrated with gold interdigitated electrodes and functionalized with anti-VEGF antibodies, enabling in situ impedimetric sensing directly in interstitial fluid. Mechanical testing confirmed robust insertion capacity, with each needle tolerating up to 0.275 N without fracture, ensuring painless and reliable skin penetration. The biosensor achieved a linear detection range of 100–1000 pg mL^−1^ with a detection limit of 100 pg mL^−1^ and a sensitivity of 0.47 nF pg^−1^ mL, covering clinically relevant VEGF concentrations. Selectivity assays demonstrated minimal interference (<2% capacitance change) from proteins such as CEA, myoglobin, and troponin I, underscoring strong specificity. Notably, antibody immobilization efficiency reached ~69%, verified by SDS-PAGE and Bradford analysis, confirming stable surface functionalization. These findings highlight the potential of MIdE MNs as minimally invasive, label-free, and real-time biosensors for monitoring VEGF in cancer, diabetic retinopathy, and cardiovascular disorders, marking an important step toward wearable protein diagnostics [128]. VEGF monitoring extends beyond cancer diagnosis to therapeutic monitoring. Anti-angiogenic drugs targeting VEGF pathways represent major oncology therapeutics, yet treatment response varies widely, and toxicities are significant. Real-time VEGF tracking could enable personalized dose optimization, early identification of treatment resistance, and prediction of adverse vascular events, transforming these therapies from fixed-dosing protocols to dynamically adjusted, patient-specific regimens.

MN-based biosensors are also being designed for oxidative stress monitoring, targeting reactive oxygen species (ROS) such as the superoxide anion. Gold MNs were modified with electrochemically reduced graphene oxide and yttrium hexacyanoferrate to enhance conductivity, while superoxide dismutase (SOD) served as the recognition element. The resulting platform achieved a wide linear detection range of 0.304–314 μM, with a detection limit of just 17 nM and sensitivity of 0.17 nA μM^−1^, effectively covering physiological and pathological superoxide levels. The device demonstrated strong selectivity, with negligible responses to common interferents (ascorbic acid, glucose, dopamine, uric acid, H_2_O_2_), and retained 85% of its activity after one month, confirming its stability. Importantly, the biosensor successfully detected superoxide release from stimulated prostate cancer (PC3) cells in real time, demonstrating its translational potential for cancer diagnostics and oxidative stress research. This MN-based system provides a minimally invasive, highly sensitive, and durable platform for monitoring ROS dynamics, offering new opportunities in both clinical oncology and redox biology [129]. ROS monitoring addresses an emerging paradigm in cancer biology; oxidative stress serves dual roles as both a driver of carcinogenesis and a vulnerability that can be therapeutically exploited. The ability to quantify tumor microenvironment ROS levels could inform treatment selection between pro-oxidant therapies (radiotherapy, certain chemotherapeutics) and antioxidant interventions, while also providing mechanistic insights into treatment resistance mechanisms mediated by altered redox homeostasis.

**Figure 17:**
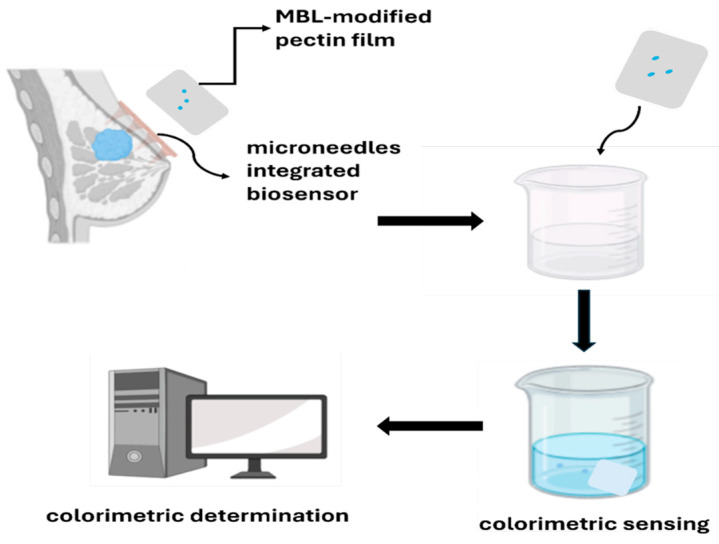
Schematic representation of early breast cancer detection using MN-assisted interstitial fluid sampling.

As the MN technologies are being explored for early cancer diagnostics, offering a painless and blood-free alternative to conventional approaches, MNs were combined with a breathable MBL thin film and a nano-Ag-based colorimetric platform (Figure 17) to detect carcinoembryonic antigen (CEA) in tissue fluid near mammary tumors, as achieved by Chen et al. [130]. In animal models, the system enabled tumor detection as early as day 8 post-inoculation, whereas standard blood tests only identified cancers on day 15, providing a 7-day diagnostic advantage. The tissue-fluid assay produced quantifiable blue color changes proportional to CEA levels, with digital image analysis yielding average color intensity values of ~164 at diagnosis, compared with <50 in healthy controls. Importantly, when doxorubicin therapy was initiated at this early stage, tumor growth was effectively suppressed, body weight stabilized, and all treated rats survived beyond 30 days, in contrast to untreated or late-diagnosed groups, which showed rapid decline and death within 3–4 weeks. Preliminary human testing in 10 volunteers confirmed feasibility, healthy subjects showed values below the diagnostic threshold (<50), while cancer patients displayed elevated responses (>100). This MN–nano-Ag/MBL system demonstrates a minimally invasive, rapid, and highly sensitive approach for early breast cancer detection, with significant implications for improving survival through timely intervention [130]. The 7-day diagnostic window represents more than a temporal advantage; it corresponds to multiple tumor doubling times during exponential growth phases and potentially precedes lymphatic or vascular invasion. This early detection capability could fundamentally alter screening strategies, rather than relying on annual mammography with inherent radiation exposure and false-positive rates, frequent or continuous tissue-fluid monitoring could enable truly pre-symptomatic detection while patients remain asymptomatic and tumors remain localized. The colorimetric readout, requiring no specialized equipment, further democratizes access, potentially enabling screening in resource-limited settings where conventional diagnostic infrastructure is unavailable.

#### 4.2.3. Infectious Diseases

Biosensor-integrated MNs are increasingly being developed for infectious disease applications. These emerging systems can detect infection-related biomarkers such as viral antigens or bacterial toxins in a minimally invasive, real-time manner by sampling interstitial fluid directly from the skin [131]. The COVID-19 pandemic starkly illustrated limitations in current infectious disease diagnostics. Nasopharyngeal swab collection requires trained personnel, generates infectious waste, causes patient discomfort that reduces compliance with repeated testing, and introduces sampling variability that affects diagnostic accuracy. MN-based pathogen detection addresses these constraints while enabling deployment scenarios, including home testing, workplace screening, and airport surveillance that conventional methods cannot readily support.

Additionally, MN platforms have also demonstrated the ability to deliver antimicrobial agents, for instance, dissolvable MNs loaded with chloramphenicol gelatin nanoparticles that automatically release the drug at infection sites upon needle dissolution. This dual functionality paves the way for closed-loop, responsive, point-of-care systems capable of on-demand detection and targeted treatment of infections [132]. This capability provides a rapid response system for treating infections, improving patient outcomes by delivering drugs at the right time and in the right amount. This detect-and-treat paradigm is particularly relevant for emerging antimicrobial resistance challenges. Conventional empirical antibiotic therapy contributes to resistance development by exposing patients to broad-spectrum agents regardless of actual infection status or pathogen identity. Biomarker-triggered antimicrobial release could enable pathogen-specific therapy initiation only when infection is confirmed, reducing unnecessary antibiotic exposure while ensuring prompt treatment when genuinely needed.

Zn-MOF-loaded MeHA MN system brings together two critical functions needed for effective wound care, strong antibacterial protection and support for tissue regeneration. The gradual release of zinc ions not only disrupts bacterial membranes and triggers oxidative stress to eliminate pathogens but also works in harmony with the hydrolyzed hyaluronic acid to encourage angiogenesis, collagen formation, and reduced inflammation. These MNs interact gently with the wound surface, providing a painless and biocompatible approach. Importantly, this platform was shown to accelerate epithelial repair and blood vessel growth, underscoring its potential as a patient-friendly strategy for faster and safer wound healing [133]. Chronic wound management represents a substantial healthcare burden, with diabetic foot ulcers alone affecting 15–25% of diabetic patients and contributing to over 70,000 annual lower-limb amputations in the United States. The dual antimicrobial-regenerative approach addresses the fundamental pathophysiology; chronic wounds persist due to both sustained bacterial colonization that prevents healing and impaired angiogenesis that deprives tissues of nutrients and oxygen necessary for repair. Simultaneous intervention on both fronts could break this pathological cycle more effectively than sequential or single-mechanism therapies. The MN-MOF-GO-Ag patch not only demonstrated superior antibacterial performance and rapid in vitro wound closure but also translated these effects into significant in vivo benefits, including faster wound contraction, enhanced tissue repair, and reduced inflammation. By uniting infection control with regenerative stimulation in a single, minimally invasive platform, this MN-based system represents a promising therapeutic advance for chronic diabetic wounds and holds strong potential for future clinical translation [134]. The incorporation of multiple antimicrobial mechanisms, metal–organic frameworks, graphene oxide, and silver nanoparticles, addresses the escalating threat of multidrug-resistant wound pathogens, particularly methicillin-resistant *Staphylococcus aureus* and *Pseudomonas aeruginosa*, which frequently colonize chronic wounds and resist conventional topical antibiotics. Multi-modal antimicrobial approaches reduce the likelihood of resistance emergence while maintaining efficacy against established resistant strains.

Cytokine profiling is critical for early diagnosis and therapeutic guidance. Cytokine dysregulation underlies the pathophysiology of severe infections, and sepsis-associated cytokine storms cause the majority of infection-related mortality not through direct pathogen effects but through excessive immune activation leading to multi-organ failure. Early detection of cytokine elevation, hours before clinical deterioration becomes apparent, could enable preemptive immunomodulatory interventions during the narrow therapeutic window when such treatments remain effective. Song et al. [135], has fused-silica MN to be integrated with a microwell impedance sensor and functionalized with antibodies for label-free, real-time cytokine detection. The device demonstrated robust mechanical strength for transdermal insertion and a broad detection range for interleukin-8 (IL-8), spanning 62 pg/mL to 539 ng/mL (R^2^ = 0.97), comfortably covering both physiological and pathological levels. In vitro assays confirmed picomolar sensitivity and high specificity, with negligible interference from non-target serum proteins. Importantly, in vivo testing in transgenic mice expressing human IL-8 showed strong agreement with ELISA, validating its quantitative accuracy in live subjects. Beyond IL-8, the platform was also adapted for IL-6 and TNF-α detection, two key mediators of infection and sepsis, highlighting its versatility. These results establish microwell-integrated MN biosensors as minimally invasive, rapid, and reliable tools for tracking cytokine storms and immune responses during infectious diseases, with significant potential for point-of-care deployment in critical care and vaccine monitoring [135]. The ability to monitor multiple cytokines simultaneously addresses a critical limitation of single-biomarker approaches. Cytokine storm syndromes involve complex, temporally dynamic patterns where IL-6, IL-8, TNF-α, and other mediators rise in characteristic sequences. Multiplexed detection enables pattern recognition that distinguishes between bacterial species, viral infections, and non-infectious inflammatory conditions, diagnostic distinctions that fundamentally alter treatment strategies but remain challenging with current rapid diagnostics.

**Figure 18:**
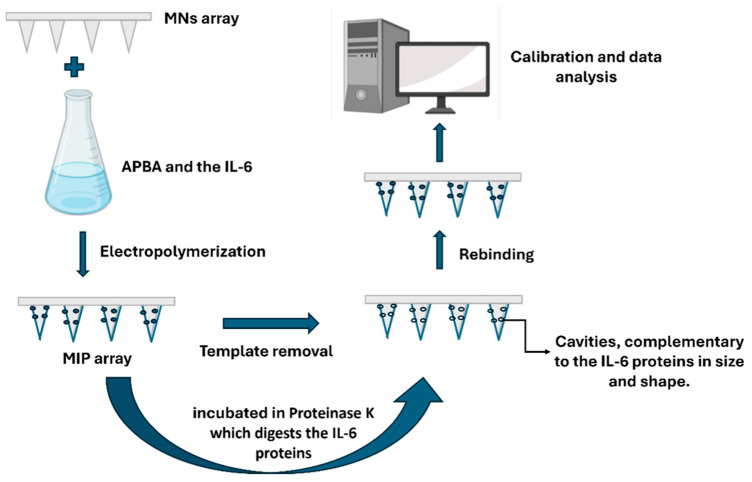
Fabrication of MIP-based sensing devices on MN array.

MN arrays combined with molecularly imprinted polymers (MIPs), as illustrated in Figure 18, are emerging as low-cost tools for monitoring inflammatory cytokines linked to infectious diseases. Polycarbonate MNs were functionalized with MIP (plastic antibodies) designed to selectively capture interleukin-6 (IL-6), a key mediator of sepsis, pneumonia, and viral infections such as COVID-19. The platform achieved rapid electrochemical readouts within 6 min, with a linear detection range from 1 pg/mL to 10 ng/mL and a limit of detection as low as 1 pg/mL in artificial interstitial fluid. Calibration curves demonstrated excellent reproducibility (R^2^ > 0.98), while non-imprinted controls failed to show similar specificity, confirming the selectivity of the imprinting approach. Importantly, the use of scalable injection-molded MNs and electropolymerized MIPs supports affordable mass production. By enabling painless, blood-free, and highly sensitive cytokine monitoring directly through the skin, this approach holds significant promise for point-of-care infectious disease diagnostics and early intervention during cytokine storm syndromes [136]. The MIP approach offers economic and logistical advantages over antibody-based recognition. Antibodies require biological production systems, cold-chain storage, and have limited shelf-life, creating supply chain vulnerabilities particularly problematic during pandemic surges when demand suddenly escalates. Synthetic MIPs remain stable at room temperature for extended periods, can be manufactured using standard polymer chemistry without biological infrastructure, and offer cost structures potentially orders of magnitude lower than antibody production, factors critical for pandemic preparedness and deployment in resource-limited settings where infectious disease burden is highest.

MN-based biosensors are proving transformative in infectious disease diagnostics, where early, sensitive, and minimally invasive testing is essential. The clinical imperative for improved diagnostics is evident in infectious disease mortality patterns, where delayed pathogen identification and treatment initiation contribute to preventable deaths in sepsis, pneumonia, and emerging viral infections, where each hour of delay in appropriate antimicrobial therapy increases mortality risk by 7–10%. Hsu et al. [137] introduced a Lab-on-the-Needles SenBox platform, integrating MN patches with horseradish peroxidase–loaded ZIF-8 probes for on-needle colorimetric analysis of protein biomarkers in saliva, sputum, and skin interstitial fluid. The system achieved ultralow detection limits of 5–10 pg/mL for anti-SARS-CoV-2 IgA and S1 spike proteins from Delta and Omicron variants, representing a 10–20-fold sensitivity improvement compared with conventional 2D assays and outperforming FDA-approved lateral flow kits by up to 1000-fold. Clinical saliva samples from COVID-19 patients were correctly identified within 30 min, with recovery rates of 97–105% and R^2^ values above 0.99, demonstrating excellent accuracy and reproducibility. The 1000-fold sensitivity advantage over approved rapid tests addresses a critical limitation exposed during the COVID-19 pandemic: lateral flow antigen tests frequently produce false negatives during early infection when viral loads are low, but patients are already contagious, the precise period when accurate detection would most effectively prevent transmission. This enhanced sensitivity could enable reliable pre-symptomatic detection that current rapid tests cannot achieve. Beyond viral detection, the SenBox was validated in a rat inflammation model, enabling real-time epidermal quantification of TNF-α and IL-1β at concentrations ranging from 10 pg/mL to 3000 pg/mL, closely matching Western blot and IHC results. The device maintained stability for 21 days at 25 °C without loss of activity, supporting practical storage and deployment. This study highlights MN SenBox platforms as powerful, portable, and patient-friendly solutions for infectious disease surveillance, cytokine storm monitoring, and rapid outbreak control [137]. The room-temperature stability eliminates cold-chain requirements that constrain conventional diagnostic deployment. Many antibody-based assays require refrigeration at 2–8 °C throughout storage and transport, creating logistical barriers in tropical climates, rural areas, and emergency response scenarios where refrigeration infrastructure is unavailable or unreliable. Ambient-stable diagnostics enable stockpiling for pandemic preparedness, field deployment during outbreaks, and point-of-care testing in primary care clinics and pharmacies lacking specialized storage facilities.

Inflammatory biomarkers such as CRP and procalcitonin (PCT) are vital for early detection of sepsis and pneumonia [138]. Sepsis represents a time-critical medical emergency where diagnostic delays directly translate to mortality. The golden hour concept in sepsis management emphasizes that survival probability decreases exponentially with time from symptom onset to appropriate antibiotic administration. Current diagnostic pathways, involving clinical suspicion, blood culture collection, laboratory transport, and culture incubation, typically require 24–72 h for definitive pathogen identification, forcing clinicians to initiate broad-spectrum empirical therapy that may be inappropriate, inadequate, or contribute to resistance development. Low-cost stereolithography 3D printing was used to fabricate hollow MN arrays (~1 mm height, ~495 μm bore, ~25 μm tip radius) capable of passive interstitial fluid extraction (~22.5–60 μL in 5 min). Surface PEGylation enhanced hydrophilicity, ensuring stable fluid flow for up to 30 days while maintaining > 85% skin cell viability, confirming biocompatibility. The MNs displayed strong mechanical integrity, penetrating porcine skin to depths of ~758 μm without damage. Integrated with commercial lateral flow assays, the platform enabled simultaneous detection of CRP and PCT at clinically relevant thresholds (CRP ≥ 10 μg/mL, PCT ≥ 1 ng/mL) within 5 min, with clear positive test lines observed. These concentrations correspond to elevated inflammatory states and correlate with early sepsis risk. Importantly, the 3D-printed design achieved a production cost of less than $0.05 per array, supporting scalability for low-resource settings. Together, these results demonstrate that 3D-printed hollow MN–LFA devices can provide fast, blood-free, and affordable point-of-care diagnostics for infectious disease-related inflammation [138]. The sub-$0.05 manufacturing cost represents a potential paradigm shift in global diagnostic accessibility. Conventional sepsis biomarker testing costs $50–200 per test in developed healthcare systems and is often entirely unavailable in low-income countries where sepsis mortality rates are highest. At this cost point, routine screening becomes economically feasible even in resource-constrained settings, potentially enabling early sepsis detection in community health centers, rural clinics, and home healthcare scenarios currently lacking access to laboratory infrastructure. Furthermore, the disposable single-use design eliminates sterilization requirements and cross-contamination risks that complicate reusable diagnostic devices in settings with limited infection control infrastructure.

**Table 2:** Microneedle-enhanced biosensors for the detection of disease biomarkers.

**Biomedical Application**	Material	**Target Biomarker**	**Recognition Strategy**	**Transduction Mechanism**	**References**
Metabolic disorder biomarkers	Silk/D-sorbitol hydrogel MN	Glucose	Glucose Oxidase (GOx)	Direct H_2_O_2_ Oxidation	[114]
	Porous gold MN	Glucose	FAD-GDH, FcSH redox mediator	Mediated electron transfer	[115]
	Flexible MN array	Glucose, Uric Acid, Cholesterol	Glucose Oxidase, Uricase, Cholesterol Oxidase	Electrochemical	[139]
	MN array with multilayer coatings (HBD/NAD^+^, chitosan, PVC)	β- hydroxybutyrate (HB), Glucose, Lactate	Glucose, Lactate	Electrochemical	[140]
	Gold-coated MN	Glucose, Lactate	Lactate Oxidase	Mediated electron transfer via pMB	[116]
	3D-printed MN array	Glucose	GOx immobilized on the electrode	Electrochemical (H_2_O_2_ oxidation)	[117]
	Gold MN	Lactate oxidase	Au multiwalled carbon nanotube, polymethylene blue (pMB) electropolymerization	Mediated electron transfer via pMB	[73]
	Solid MN	Lactate oxidase (LOx) immobilized in a hydrogel	Coupled to a potentiostat	Electrochemical (H_2_O_2_ Oxidation)	[141]
	Gold-plated PLA MN	Cortisol	Cortisol, a specific DNA aptamer immobilized on AuNPs	Electrochemical	[142]
	Swellable methylacrylate hyaluronic acid MN	Cortisol	Aptamer–C duplex dissociation upon cortisol binding, initiating HCR (H1/H2 hairpins)	Fluorescence (FAM signal amplification)	[143]
	Hollow MN with flexible microfluidic chip	Uric acid (UA)	Label-free (direct detection, no enzymes/aptamers)	SERS	[118]
	Solid gold-plated MN array	Lactate oxidase	Gold-plated working electrode, Polyaniline for conductivity, Nafion for selectivity	Enzymatic oxidation of lactate	[119]
Infectious disease biomarkers	Fused silica MN with microwells	Human Interleukin- 8 (hIL8)	Anti-hIL8 antibodies immobilized inside microwells	Electrochemical impedance spectroscopy (EIS)	[135]
	Platinum-coated MN array with molecularly imprinted polymer (MIP) layer	Interleukin- 6 (IL-6)	MIP cavities formed via APBA electropolymerization	EIS with [Fe(CN)6]^3−^/^4−^ redox probe	[136]
	Gold nanoparticle (AuNP)-coated MN patch (SenBox platform)	TNF-α, Interleukin- 1β (IL-1β)	Antibodies conjugated to AuNP-coated MN capture cytokines, and HRP@ZIF-8 is used for signal amplification	Colorimetric	[137]
	3D-printed PEGylated hollow MN	Procalcitoni n (PCT), CRP	Antibody–antigen binding on LFA strip (Au-conjugates)	Colorimetric	[138]
Cancer biomarkers	High-density gold-coated silicon MN array (Au–Si-MNA)	ErbB2 (HER2)	Anti-HER2 antibodies immobilized on Au–Si-MNA	Electrochemical	[144]
	Polylactide (PLA) MNs with interdigitated electrodes (MIdE)	VEGF	Anti-VEGF antibodies	Capacitive biosensing	[128]
	Gold MN (AuMN) modified with reduced graphene oxide (rGO), yttrium hexacyanoferrate (YHCF), and immobilized superoxide dismutase (SOD)	Superoxide anion	Enzymatic redox cycling via Cu–Zn SOD; superoxide undergoes sequential enzymatic reactions leading to a current drop	Electrochemical	[129]
	MNassisted extraction with Ag_3_PO_4_@Ag magnetic nanoparticles and Ag_3_PO_4_/Ag nanocomposites	Carcinoembryonic antigen (CEA)	Primary antibodies on Ag_3_PO_4_@Ag capture CEA, secondary antibodies on Ag_3_PO_4_/Ag act as catalytic labels	Colorimetric assay	[130]
Cardiovascular disease biomarkers	Au-coated PLA arrayed MNs	CRP	ssDNA aptamer immobilized via thiol-gold chemistry	Colorimetric enzyme-linked assay	[122]
	3D-printed pyramidal MN array with platinum and silver electrodes	Cholesterol	Cholesterol oxidase (ChOx) immobilized via Nafion, BSA matrix	Electrochemical Chronoamperometry	[123]
	Hydrogel MNs with a graphene working electrode and PANI interlayer	Cholesterol	Cholesterol oxidase (ChOx) immobilized on PANI modified graphene electrode	Cyclic voltammetry	[124]

### 4.3. Current Status and Emerging Trends

The field of biosensor-integrated MNs has reached a stage of rapid technological maturation, transitioning from proof-of-concept demonstrations toward clinically relevant diagnostic and therapeutic platforms. This section provides an overview of the present state of development of MN-based biosensor technologies while highlighting key emerging trends that are expected to influence their future advancement.

At present, electrochemical MN biosensors for glucose and metabolic monitoring represent the most advanced and widely investigated systems, with several devices demonstrating robust performance, miniaturization, and integration into wearable formats [13,69,94]. Advances in MN fabrication, materials engineering, and interstitial fluid sampling have established a solid technological foundation for minimally invasive, real-time biomarker monitoring [18,23,95].

Several clear trends are shaping the current trajectory of the field. First, there is a strong shift toward continuous and real-time monitoring using wearable MN platforms, particularly for chronic diseases such as diabetes and cardiovascular disorders [73,106,114]. Second, MN systems are evolving from passive delivery devices into smart, closed-loop platforms capable of biomarker-guided and autonomous drug release [75,94,102]. Third, the integration of artificial intelligence, Internet of Things connectivity, and cloud-based analytics is increasingly enabling personalized, data-driven healthcare, positioning MN biosensors as key components of future digital health ecosystems [70,145,146]. Finally, the scope of detectable biomarkers is expanding beyond glucose to include lactate, cortisol, inflammatory cytokines, infectious disease markers, and cancer-associated proteins, reflecting a move toward multiplexed and systems-level physiological monitoring [69,94,125].

Despite these advances, several challenges continue to impede widespread clinical translation. Manufacturing scalability and reproducibility remain major barriers, particularly for complex MN architectures requiring high precision and consistent mechanical performance [147,148]. Material-related challenges, including insufficient mechanical strength in dissolving and hydrogel-forming MNs, blockage or leakage in hollow MNs, and long-term storage stability, require further optimization to ensure reliable real-world use [34,40,43]. From a biosensing perspective, enzyme instability, biofouling, signal drift, and interference from the complex biochemical environment of the skin remain critical issues, particularly for long-term continuous monitoring applications [149,150]. In addition, regulatory approval, large-scale clinical validation, and seamless integration into existing healthcare workflows represent substantial non-technical challenges that must be addressed before biosensor-enhanced MN systems can achieve routine clinical deployment [95,102,151].

Although many MN-based biosensors show excellent performance in laboratory studies, translating these systems into clinical practice requires overcoming challenges beyond sensitivity and selectivity. Successful platforms typically rely on biocompatible materials, stable recognition elements, and reliable sensing interfaces that can function effectively within the complex environment of interstitial fluid [152,153]. However, several prototypes remain limited by issues such as signal drift, biofouling, and the long-term stability of sensing components. In addition, practical factors—including scalable manufacturing, regulatory approval, and patient usability—play a crucial role in determining whether these technologies can move from the laboratory to real-world healthcare applications [154,155]. The future success of MN biosensors will depend on balancing technological innovation with practical implementation to ensure reliable and clinically relevant performance.

## 5. Challenges and Limitations

Despite the promising potential of MN-biosensor systems in drug delivery, several challenges and limitations must be addressed for their widespread clinical application. These challenges range from technical and engineering issues to the extent of biocompatibility and regulatory concerns. This section examines the principal technical, biological, and regulatory challenges that presently hinder the broad clinical translation of MN–biosensor systems. It aims to critically delineate the key barriers that must be overcome to achieve device safety, reliability, scalability, and successful regulatory approval.

The gap between laboratory demonstration and clinical implementation often spans years or decades, with many promising technologies failing to translate due to unforeseen complications in real-world deployment, regulatory requirements exceeding initial expectations, or manufacturing costs proving commercially prohibitive. In this section, we will explore the primary obstacles faced by these systems, including miniaturisation, signal interference, safety concerns, and regulatory hurdles.

### 5.1. Biocompatibility

For MN-biosensor systems to be safe and effective, they must be biocompatible, meaning they do not elicit an immune response or cause tissue damage. Materials used in both the MNs and the biosensors must be nontoxic and compatible with the skin. Biocompatibility assessment extends beyond acute toxicity testing to encompass chronic inflammatory responses, sensitization potential, and interactions with compromised skin barriers, considerations particularly critical for patients with dermatological conditions, immunosuppression, or the elderly with fragile skin who represent key target populations for continuous monitoring devices.

One challenge with silicon-based MNs is their rigidity, which can cause microtears or local irritation when inserted into the skin [156]. Although biodegradable materials such as polylactic acid (PLA) and polyvinyl alcohol (PVA) are commonly used for dissolving MNs, these materials must degrade at a controlled rate to avoid leaving harmful residues [157]. Degradation kinetics must be precisely matched to application requirements, too rapid and mechanical integrity fails before drug delivery completes or biosensing concludes; too slow and residual polymers accumulate in tissue, potentially triggering foreign body responses or creating depots that complicate repeated application at the same site, a concern for patients requiring daily or continuous monitoring.

The integration of biosensors into MN systems can provoke immune responses if the components are fabricated from non-biocompatible materials, particularly certain metals or synthetic polymers. This hypersensitivity may manifest as localized inflammation, allergic reactions, or even tissue damage, undermining the system’s effectiveness and patient safety [158]. Metal allergenicity presents particular challenges; nickel sensitivity affects 10–20% of the population, while other common electrode materials, including chromium and cobalt, also demonstrate significant sensitization rates. These prevalence rates mean that either allergenic materials must be avoided or patients must be pre-screened, both of which increase cost and complexity and can limit commercial feasibility.

MNs are designed for short-term use, but as their applications expand, there may be concerns about their long-term safety. In particular, the long-term effects of having MN residues or biosensor by-products in the skin or body are not yet fully understood. This raises questions about the degradation of the materials and whether they can be safely absorbed or excreted [159]. Chronic implantation introduces additional considerations beyond acute biocompatibility, fibrotic encapsulation, progressive biofouling that degrades sensor performance, potential for nanomaterial accumulation in regional lymph nodes or systemic circulation, and the possibility of late-onset hypersensitivity reactions developing after months or years of exposure, outcomes that short-term preclinical studies cannot adequately predict.

Long-term studies and clinical trials will be needed to evaluate the chronic safety of these systems, especially when used for continuous monitoring or repeated applications. Regulatory pathways for long-term implantable or repeatedly applied devices require extensive safety data, typically 1–2 year animal studies, followed by phased human trials with extended follow-up periods. This timeline, combined with the substantial financial investment required, often $50–100 million for a novel medical device seeking regulatory approval, creates significant barriers that small companies and academic groups struggle to overcome, potentially slowing innovation in this field.

### 5.2. Miniaturization

A major challenge in advancing MN-biosensor technology lies in the demand for extreme miniaturization; MNs must be sufficiently small to minimize insertion pain while also being large enough to house integrated biosensors and, in some designs, drug reservoirs. Achieving an optimal balance between physical dimensions, mechanical durability, and functional capacity remains a formidable engineering challenge [147]. This dimensional constraint creates competing requirements; reducing needle diameter from 300 to 150 micrometers may halve the pain perception, but it also reduces the available surface area for enzyme immobilization by 75%, potentially decreasing sensor sensitivity below clinically useful thresholds unless compensatory signal amplification strategies are implemented.

As MNs decrease in size to minimize pain during insertion, they risk losing the mechanical strength necessary to reliably penetrate the stratum corneum, particularly when constructed from biodegradable polymers. This reduction in structural robustness can compromise the device’s ability to create stable skin microchannels, thereby limiting consistent biomarker access or drug delivery [148]. Flexible MNs fabricated from biodegradable materials may provide a promising solution to penetration limitations, offering improved skin conformity. However, this flexibility typically comes at the cost of reduced mechanical strength, which may compromise reliable skin penetration and structural stability [34]. Penetration failure rates become clinically significant issues if 5–10% of MNs in an array fail to penetrate adequately, biosensor readings become unreliable, and drug delivery is incomplete. For home-use devices where professional oversight is absent, such failure rates translate to patient frustration, treatment non-compliance, and potential adverse outcomes from inadequate therapy, problems that could undermine clinical adoption despite otherwise promising performance characteristics.

Incorporating biosensors into compact MN systems presents a formidable challenge, while microfabrication techniques like 3D printing offer the precision and versatility needed to create multifunctional MNs, ensuring the reliability and durability of such devices remains a critical hurdle. The fabrication process must deliver structural integrity, consistent biosensor performance, and long-term stability under physiological conditions, which current methods are still striving to fully achieve [95]. Manufacturing reproducibility presents particular challenges at the microscale, achieving a coefficient of variation below 10% for critical dimensions, ensuring uniform enzyme loading across thousands of MNs in a single patch, and maintaining consistent electrochemical properties despite inevitable material and process variations all require sophisticated quality control systems that increase production costs and complexity, factors that affect commercial scalability and ultimate affordability.

### 5.3. Signal Interference

Another significant challenge lies in mitigating signal interference. Biosensor performance within MN systems can be impaired by biological noise, such as ions, proteins, and other endogenous molecules present in the skin environment, which may obscure or distort biomarker detection signals [151]. Interstitial fluid composition varies substantially across individuals and physiological states. Exercise alters lactate and pH; meals affect glucose and lipids; inflammation elevates proteins and cytokines; and medications introduce metabolites. This biological variability means biosensors must maintain specificity and accuracy across a far wider range of conditions than controlled laboratory environments, requiring robust calibration strategies and interference-rejection mechanisms.

For instance, in electrochemical biosensors, the presence of electrolytes or non-target molecules can cause false positives or inaccurate readings. This can lead to erroneous drug release, which could be dangerous for patients. To mitigate this, researchers have focused on improving the selectivity of the biosensors through surface modification techniques [149]. The clinical consequences of false positives vary dramatically by application; erroneous insulin release triggering hypoglycemia can cause acute neurological injury or death; and inappropriate chemotherapy dosing from false tumor biomarker readings could either undertreat cancer or cause severe toxicity. These high-stakes scenarios demand near-perfect specificity, typically >99%, that remains challenging to achieve consistently in complex biological matrices.

Additionally, optical biosensors integrated into MN systems are particularly vulnerable to signal degradation caused by light scattering and absorption within skin tissues. These optical interferences stemming from the skin’s heterogeneous composition and intrinsic optical properties can significantly diminish the quality of fluorescent signals or light transmission, thereby impairing sensor sensitivity and accuracy [160]. Skin optical properties vary substantially with melanin content, creating performance disparities across different ethnic populations. Sensors optimized for lightly pigmented skin may show 50–80% signal attenuation in darkly pigmented skin, potentially creating health equity issues where device performance varies systematically with race. Ensuring equitable performance across diverse populations requires deliberate, inclusive design and validation across representative demographic groups, considerations often inadequately addressed in early-stage development.

### 5.4. Molecular Recognition as the Primary Bottleneck

Although considerable progress has been made in the engineering design and microfabrication of microneedle (MN) arrays, the major challenge limiting the advancement of next-generation MN biosensors increasingly lies in the biochemical recognition elements responsible for analyte detection. In most MN-based sensing platforms, analytical performance is largely dictated by the affinity, selectivity, and stability of the recognition interface rather than by the physical MN structure itself. Consequently, even highly optimized MN architectures may fail to achieve reliable sensing if the molecular recognition layer is unstable or exhibits insufficient specificity within the complex biological environment of interstitial fluid (ISF). Glucose monitoring represents the most successful example of MN-integrated biosensing, largely due to the well-characterized enzymatic reaction catalyzed by glucose oxidase (GOx). The robustness, specificity, and long-term stability of GOx have enabled the commercialization of several continuous glucose monitoring systems and have served as a model for enzymatic biosensing technologies. However, extending MN-based sensing platforms beyond glucose to other clinically relevant biomarkers remains significantly more challenging. Biomolecules such as cytokines, therapeutic drugs, hormones, metabolic intermediates, and cancer biomarkers often occur at very low concentrations in ISF, may undergo rapid degradation, and frequently require highly selective recognition mechanisms to avoid cross-reactivity with structurally similar molecules [161,162]. To address these challenges, various biorecognition elements have been explored for integration into microneedle biosensors, including antibodies, aptamers, enzymes, and molecularly imprinted polymers (MIPs). Each of these recognition strategies presents distinct advantages and limitations. Antibodies offer excellent specificity but may be structurally unstable and costly to produce. Aptamers provide strong binding affinity and can be chemically synthesized with high reproducibility, yet they may experience conformational instability under physiological conditions. Enzyme-based systems offer catalytic signal amplification but are often sensitive to temperature and pH fluctuations. Meanwhile, MIPs provide superior chemical stability and reusability but may exhibit lower binding specificity compared with biological recognition molecules [163,164].

A critical requirement for these recognition elements is their ability to retain structural integrity and functional activity in the chemically complex interstitial fluid, which contains proteins, salts, metabolites, and enzymes that can interfere with sensing performance. In addition, long-term monitoring applications demand that recognition layers resist biofouling, degradation, and signal drift during continuous operation. These factors collectively contribute to reduced sensitivity and measurement reliability over extended wear periods, representing a key barrier to the clinical translation of multiplexed MN biosensing systems [154,162].

Therefore, improving molecular recognition strategies through advanced surface functionalization, antifouling coatings, engineered biomolecules, and hybrid synthetic–biological receptors has become a central research focus in the field of wearable microneedle biosensors. Continued progress in this area is expected to enable the detection of a broader range of clinically important biomarkers and facilitate the transition of microneedle sensing technologies from proof-of-concept laboratory devices to robust platforms for continuous health monitoring and personalized medicine.

### 5.5. Translational and Commercial Barriers

Although MN technologies have demonstrated impressive performance in laboratory and preclinical studies, their transition from research prototypes to widely adopted commercial products has proven to be considerably more complex. Many MN systems show excellent mechanical performance, precise skin penetration, and promising biosensing or drug-delivery capabilities under controlled experimental conditions. However, translating these innovations into scalable, regulatory-approved medical devices requires overcoming practical challenges that extend beyond the laboratory. These include large-scale manufacturing, regulatory compliance, cost-effectiveness, long-term device stability, and user acceptance, all of which play a decisive role in determining whether a technology can successfully reach the healthcare market [165,166]. Historically, even technically robust microneedle products have struggled to achieve widespread commercialization. A commonly cited example is the microneedle drug-delivery patch developed by 3M, which demonstrated strong engineering performance but did not reach broad market adoption. One of the key reasons for this difficulty is the challenge of scaling up microfabrication techniques while maintaining uniform needle geometry, mechanical strength, and sterility at industrial production levels. In addition, MN devices must satisfy stringent regulatory requirements imposed by agencies such as the U.S. Food and Drug Administration (FDA) and the European Medicines Agency (EMA). These regulatory pathways often require extensive safety validation, biocompatibility testing, and clinical trials, which significantly increase development time and cost [162,166,167].

Cost considerations and patient acceptance also influence commercial viability. Although microneedles are designed to provide minimally invasive and pain-free alternatives to conventional injections, patient perception of skin-penetrating devices and concerns regarding self-administration may still affect adoption rates. Moreover, manufacturing costs must remain competitive with existing technologies, such as conventional transdermal patches or blood-based diagnostic assays in order for healthcare providers and insurers to support widespread implementation [168].

## 6. Future Directions

As MN-based biosensor systems advance, new developments in their technology, design, and practical use are emerging. These innovations are designed to address current limitations and enhance the flexibility and capabilities of the sensors, allowing them to be applied across a wider range of medical needs. The convergence of multiple technological advances, improved nanomaterials, AI-driven analytics, wireless connectivity, and miniaturized electronics is creating opportunities that were technologically infeasible even five years ago, suggesting we may be approaching inflection points where laboratory innovations begin translating into clinically deployed systems at scale.

The future of MN-enhanced biosensor systems is moving toward their smooth integration with wearable technologies, creating new possibilities for continuous health monitoring and controlled drug delivery. When MN arrays are built into everyday devices like wristbands or skin patches, they can track biomarkers around the clock and deliver treatment with remarkable precision. This approach holds promise for chronic conditions such as diabetes, heart disease, and asthma, where real-time feedback can make a life-changing difference based on the patient’s changing needs, making it a truly personalized healthcare experience [151,169,170]. Wearable integration introduces human factors considerations beyond technical performance; devices must be comfortable enough for 24/7 wear, aesthetically acceptable across diverse cultural contexts, waterproof for showering and swimming, and durable enough to withstand normal physical activity. These practical requirements often prove as challenging as the underlying biosensor technology and significantly influence user acceptance and long-term adherence.

Artificial intelligence (AI) and machine learning (ML) are set to become key tools for the interpretation of the complex data collected by biosensors. By analyzing these continuous data streams, AI and ML can identify patterns, predict potential health issues, and provide real-time support for clinical decision-making [145]. AI integration enables capabilities impossible with simple threshold-based alerts, detecting subtle multi-parameter patterns indicating impending diabetic ketoacidosis hours before clinical symptoms, distinguishing between normal physiological variation and pathological changes requiring intervention, and personalizing therapeutic algorithms to individual response patterns learned from historical data, transforming raw biosensor signals into actionable clinical intelligence.

AI algorithms combine historical information with real-time data from wearable biosensors to track health outcomes and anticipate future conditions. For instance, devices that monitor heart rate variability can predict potential heart problems, allowing for early detection and timely medical intervention [146]. Predictive algorithms face substantial validation challenges; accurately forecasting adverse events hours or days in advance requires demonstrating both sensitivity (capturing most true events) and specificity (minimizing false alarms that cause alarm fatigue and healthcare system burden). Achieving this balance across diverse patient populations with varying baseline risks requires extensive training datasets and rigorous clinical validation, requirements that extend development timelines but are essential for safe clinical deployment.

ML can enhance continuous glucose monitors by predicting fluctuations in blood sugar levels based on a person’s diet and physical activity. This allows individuals with diabetes to receive more personalized and proactive guidance. Additionally, ML helps in the interpretation of biological data by filtering out background noise and highlighting meaningful patterns [150]. This approach can improve healthcare decisions by enabling more accurate interpretation of biomarkers in noninvasive samples such as sweat or saliva [171]. However, ML algorithm opacity presents regulatory and clinical acceptance challenges, black box decision-making that cannot be explained to clinicians or patients raises concerns about trust, accountability when errors occur, and the ability to identify and correct systematic biases. Developing explainable AI approaches that provide interpretable rationales for predictions represents an important research direction for clinical translation. Moreover, ML algorithms have been used to analyze electrochemical signals from wearable microneedle sensors to improve calibration, reduce noise interference, and predict glucose trends during continuous monitoring [172]. In addition, deep learning models can assist in interpreting complex biosensor datasets and correlating biomarker fluctuations with physiological conditions, supporting more accurate and personalized diagnostic decision-making [172,173].

Despite the significant potential of MN-enhanced biosensor systems, future studies need to tackle several key challenges, include the lag between biomarker levels in ISF and those in the bloodstream, which can affect the accuracy of real-time monitoring, the biological variability in skin properties and ISF dynamics from person to person, and the issue of interference among multiple analytes when detecting several biomarkers simultaneously in multiplexed systems [174]. ISF-blood lag times vary by biomarker (2–5 min for glucose, potentially longer for larger proteins), physiological state (perfusion, hydration), and anatomical site, introducing uncertainty that must be addressed through calibration algorithms or direct acknowledgment of measurement limitations. For time-critical applications like hypoglycemia prevention, even 5 min delays may compromise safety, necessitating predictive algorithms that anticipate trends rather than simply reporting current values.

Overall, MN-enhanced biosensor systems hold the potential to make diagnostic testing more accessible, promote proactive health management, and move healthcare toward a more decentralized and preventive model. This paradigm shift from episodic clinic-based care to continuous patient-centered monitoring aligns with broader healthcare trends toward value-based care, chronic disease prevention, and patient empowerment, potentially reducing healthcare costs through early intervention while improving patient quality of life. However, realizing this vision requires not only technological maturation but also addressing reimbursement models, data privacy frameworks, clinical workflow integration, and health literacy to ensure technologies serve rather than widen existing health disparities.

## 7. Conclusions

Biosensor-integrated MN systems are revolutionizing healthcare through minimally invasive biomarker sampling with high detection sensitivity. Advances in materials like hydrogels, conductive polymers, and nanocomposites have significantly improved their performance, enabling detection limits in the picogram range and long-term stability, which are crucial for clinical applications. By leveraging interstitial fluid (ISF) sampling and nanoscale signal transduction, these systems enable multiplexed detection of biomarkers associated with metabolic, inflammatory, cardiovascular, and cancerous conditions, thereby reducing patient discomfort and democratising access to continuous monitoring. This transformation allows for effective outpatient chronic disease management. The integration of artificial intelligence (AI) elevates these devices from simple diagnostic tools to intelligent health companions, supporting continuous monitoring and predictive healthcare. These capabilities address the limitations of traditional reactive healthcare models. As technical, regulatory, and implementation hurdles remain, ongoing advancements and growing clinical evidence suggest that MN biosensors are moving toward practical clinical applications. Ultimately, these technologies hold the potential to redefine disease diagnosis and therapy, promoting continuous, personalized healthcare while ensuring accessibility and equity for diverse global populations.

## Data Availability

No new data were created or analyzed in this study.
